# Caver Web 1.0: identification of tunnels and channels in proteins and analysis of ligand transport

**DOI:** 10.1093/nar/gkz378

**Published:** 2019-05-22

**Authors:** Jan Stourac, Ondrej Vavra, Piia Kokkonen, Jiri Filipovic, Gaspar Pinto, Jan Brezovsky, Jiri Damborsky, David Bednar

**Affiliations:** 1 Loschmidt Laboratories, Department of Experimental Biology and RECETOX, Faculty of Science, Masaryk University, Brno, Czech Republic; 2 International Centre for Clinical Research, St. Anne's University Hospital Brno, Brno, Czech Republic; 3 Institute of Computer Science, Masaryk University, Brno, Czech Republic

## Abstract

Caver Web 1.0 is a web server for comprehensive analysis of protein tunnels and channels, and study of the ligands’ transport through these transport pathways. Caver Web is the first interactive tool allowing both the analyses within a single graphical user interface. The server is built on top of the abundantly used tunnel detection tool Caver 3.02 and CaverDock 1.0 enabling the study of the ligand transport. The program is easy-to-use as the only required inputs are a protein structure for a tunnel identification and a list of ligands for the transport analysis. The automated guidance procedures assist the users to set up the calculation in a way to obtain biologically relevant results. The identified tunnels, their properties, energy profiles and trajectories for ligands’ passages can be calculated and visualized. The tool is very fast (2–20 min per job) and is applicable even for virtual screening purposes. Its simple setup and comprehensive graphical user interface make the tool accessible for a broad scientific community. The server is freely available at https://loschmidt.chemi.muni.cz/caverweb.

## INTRODUCTION

Proteins are biomolecules responsible for a vast variety of functions in all living organisms. They serve as a building material of cells and participate in regulation, signalling, transport, and enzymatic catalysis of small molecules. From the structural point of view, proteins consist of one or more peptide chains forming highly complex 3D structures containing many internal clefts, grooves, protrusions and voids (1). Even though such empty spaces are disadvantageous from the stability point of view, in many proteins they form functionally important local substructures, such as active sites, binding sites, allosteric sites, tunnels and channels (2, 3). Anatomies and properties of these substructures significantly influence protein functions (3). In this study, we are interested in transport pathways for small ligands represented by protein tunnels and channels. The channels are typically characterized by two openings connecting different cellular environments and play a key role in the transport of various ions and small molecules through biomembranes. The tunnels are mainly present in globular proteins with catalytic function (enzymes) and serve as the access pathways for substrates, products, co-factors, water molecules and/or inhibitors from a bulk solvent to buried active sites. They can also connect two distinct active sites within a single protein. It has been experimentally demonstrated that the tunnels and their properties can define many important protein characteristics like substrate specificity, enantioselectivity, stability and activity (4–8). Therefore, the understanding of the transport pathways, their properties and impact on ligands’ passage is important for deciphering the protein function as well as for practical applications in the fields of protein engineering and drug design.

The study of access pathways and ligand transport processes using experimental techniques is far from trivial. A quantitative description of these processes is usually obtained indirectly using transient kinetic measurements. The few available direct methods such as time-resolved crystallography and crystallography under xenon pressure are time-demanding and provide only specific information (9,10). Therefore, the function of tunnels and channels are often studied *in silico*. The tunnel and channel detection is already well a developed field (11–14). Most of the recent tools, for example Caver 3.02 (15), MolAxis 1.0 (16), Mole 2.0 (17), are based on the pathway detection in the Voronoi diagram representation of a protein structure and offer high-quality results in short calculation time.

*In silico* analyses of ligand transport are challenging and the majority of methods are based on some implementation of molecular dynamics simulations (18–21). These implementations employ various enhanced sampling approaches like Random Accelerated Molecular Dynamics (22), Steered Molecular Dynamics (23–25), Umbrella Sampling (26), Adaptive Sampling (27) or Metadynamics (26,28) and provide highly robust and accurate results. However, they are very time demanding, which prevents their usage in comparative studies or screening campaigns. Moreover, they usually require advanced knowledge of the modelling technique and a good understanding of the studied system. As an alternative, less accurate, but dramatically faster methods were developed. CaverDock 1.0 (29) and SLITHER 1.0 (30) are based on the iterative molecular docking along the tunnel, while MoMA-LigPath 1.0 (31,32) uses a robotic Manhattan-like RRT algorithm.

Here we present Caver Web 1.0, a novel web server for detection and comprehensive analysis of tunnels and channels in the protein structures. The server relies on the calculation of well-established and widely used tunnel detection software Caver 3.02. Moreover, Caver Web also integrates an explicit analysis of ligand transport through tunnels, which extends its use towards comparative studies and virtual screenings. The analysis of ligand transport is carried out by CaverDock 1.0, which provides a good trade-off between computation time and accuracy, while maintaining robustness of the workflow. A great care has been devoted to making the graphical user interface of Caver Web intuitive. The overall workflow is facilitated by robust default values of parameters and several automatic guiding mechanisms, which assist the users to correctly set up the calculation. Important results can be analysed and viewed directly in the visualization window. Three detailed tutorials cover typical use-cases, illustrating applicability of the tool for users with no prior knowledge of bioinformatics.

## WORKFLOW

**Figure 1.**
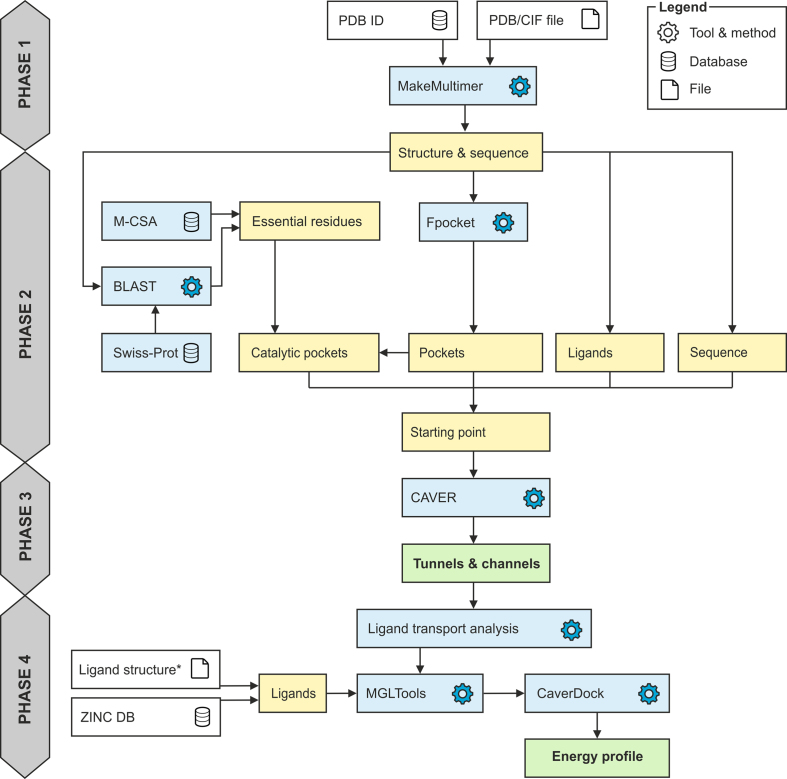
Workflow diagram of Caver Web 1.0. The process consists of four phases: (1) structure pre-treatment, (2) detection of the starting point, (3) identification of tunnels/channels and (4) analysis of ligand transport. *Ligand structures can be uploaded in all formats supported by Open Babel (http://openbabel.org/docs/current/FileFormats/Overview.html).

The basic workflow of the Caver Web tool is depicted in Figure 1. The first step of the calculation is the selection of a protein structure and its pre-treatment. The second step is a selection of a starting point for tunnel detection. Protein tunnels are identified and analysed in the third step, and optionally used to study the transport of selected ligand(s) in the fourth step.

### Structure selection and pre-treatment

The only required input is a protein tertiary structure. It can be specified either by the Protein Data Bank (33) accession code or uploaded as a file in the PDB or the CIF format. Uploaded structures are automatically converted to PDB using RCSB MAXIT tool (https://sw-tools.rcsb.org/apps/MAXIT/index.html), since Caver does not natively support CIF format. The structures are usually deposited in the form of asymmetric units, which may not reflect their naturally occurring quaternary forms (biological units) and an analysis carried on this structure may lead to wrong results and even detection of non-existing tunnels. To overcome this problem, MakeMultimer (http://watcut.uwaterloo.ca/tools/makemultimer/) is automatically executed for uploaded structures to detect their biological units. Their list and description are provided to users who can select the most appropriate biological unit or dismiss them and continue with the original structure.

### Starting point selection

The most critical step in tunnel detection is the selection of a proper starting point. The position of this point constraints the Caver calculation and defines a common starting point for all detected tunnels. A wrongly positioned point can significantly affect the relevance of detected tunnels and even lead to irrelevant tunnels. To facilitate this selection, we designed several automated protocols that provide reliable starting points suitable for the most common scenarios. In enzymes, users are often interested in access pathways for ligands leading to active or binding sites. Thus, the best starting point for this analysis is usually placed inside the pocket containing the essential residues (catalytic pocket). Since there are many tools for pocket detection and several databases of essential residues, we implemented a fully automatic ‘Catalytic pocket’ mode, which combines pockets detection with the analysis of essential residues. Pockets are detected using Fpocket 2 (34), based on the search of alpha spheres in a Voronoi tessellation representation of protein structures and subsequent clustering of the spheres to larger elements. The advantage of this tool is that it provides a druggability score, which represents a likelihood that the drug-like molecules can bind to the pocket. The essential residues are obtained from the Mechanism and Catalytic Site Atlas (35) and SwissProt (36) databases. The entries in Mechanism and Catalytic Site Atlas are mapped using the PDB accession codes. The manually curated SwissProt database is searched using BLAST with the requirement of 30% sequence identity and sequence length between 90 and 110%. After essential residues are identified, the pockets are matched with these residues and the pockets containing at least one catalytic residue are marked as catalytic. If essential residues are missing, Caver Web offers two alternative helper modes. The first one lists all detected pockets and sorts them by the estimated druggability score. The second one places the starting point to the centre of mass of any ligand present in the structure. However, this mode requires that the protein was co-crystallized or soaked with ligands, which occupy the functional site of the protein. This mode should be used with a great care. Finally, Caver Web offers the possibility to calculate the position of the starting point based on the residues selected by the user in the protein sequence, which can be further adjusted by the manual optimization of coordinates.

### Tunnel detection and analysis

Tunnel detection is carried out by Caver 3.02 (15) which searches for the paths with the given minimal radius and the lowest cost in the Voronoi tessellation representation of protein structures using Djikstra's algorithm and calculates their geometries, statistical properties and list of residues lining the tunnel and forming the bottleneck. Users can modify several important configuration parameters affecting the properties of the detected tunnels: (i) ‘residues considered for tunnel calculation’ are the parts of the structure which Caver will consider for the analysis to allow exclusion of the ligands, ions and water molecules; (ii) ‘minimum probe radius’ defines the minimal size of a spherical probe which must fit into the tunnel to be detected; (iii) ‘shell depth’ specifies the maximal depth of a surface region, i.e. shallow vertices, preventing unnecessary tunnel branching; (iv) ‘shell radius’ specifies the radius of the probe used to define which parts of the Voronoi diagram represent the bulk solvent; (v) ‘clustering threshold’ defines the similarity level at which the tunnels will be considered the same and clustered together; (vi) ‘maximal distance’ which limits how far the starting Voronoi vertex can be from the starting point position selected by the user and (vii) ‘desired radius’ which specifies how far the starting point vertex must be from the atoms of the protein structure.

### Ligand transport analysis

The last [optional] step of the workflow is the analysis of ligands transport through the detected tunnels using the CaverDock software. Initially, one or more small molecules must be provided by the user. Secondly, one or more identified tunnels are selected as the path for the ligand transport and a calculation is initiated. Caver Web adds Gastaiger charges and AutoDock Vina (37,38) compatible atom types to every atom using prepare_ligand4.py and prepare_receptor4.py scripts from the MGLTools (37) package. Then the Discretizer (29) is used to cut the tunnel to discrete slices with specified distances. Next, the CaverDock is executed to perform an iterative docking of the ligand to every slice of the tunnel using a spatially restrained AutoDock Vina docking algorithm.

Users can modify two most important parameters: (i) ‘discretization delta’ defines the distance between centres of two slices of the tunnel and (ii) ‘calculation mode’ of CaverDock defines which ligand restraints will be enforced. The first mode is called lower-bound and it enforces only the spatial restraint. This mode is very fast, however, it can miss some of the bottlenecks due to the possibility of ligand flipping, resulting in non-continuous movement. The second mode is called upper-bound and employs also the maximal ligand rotation restriction coupled with backtracking to guarantee continuous movement. Even though the continuous movements are more realistic, the analysis is computationally much more intensive and due to the limited capability of the backtracking it can overestimate energies or even completely fail to find any possible path. Therefore, the lower-bound trajectory is set as a default and users are strongly advised to use energetic profiles calculated in this mode. CaverDock supports flexible sidechains of selected residues. However, it has been shown that the energies of barriers are often artificially flattened (29), making the results difficult to interpret. For this reason, we suppressed the flexibility support in Caver Web until this issue is better resolved in future versions of CaverDock.

## DESCRIPTION OF THE WEB SERVER

### Input

**Figure 2.**
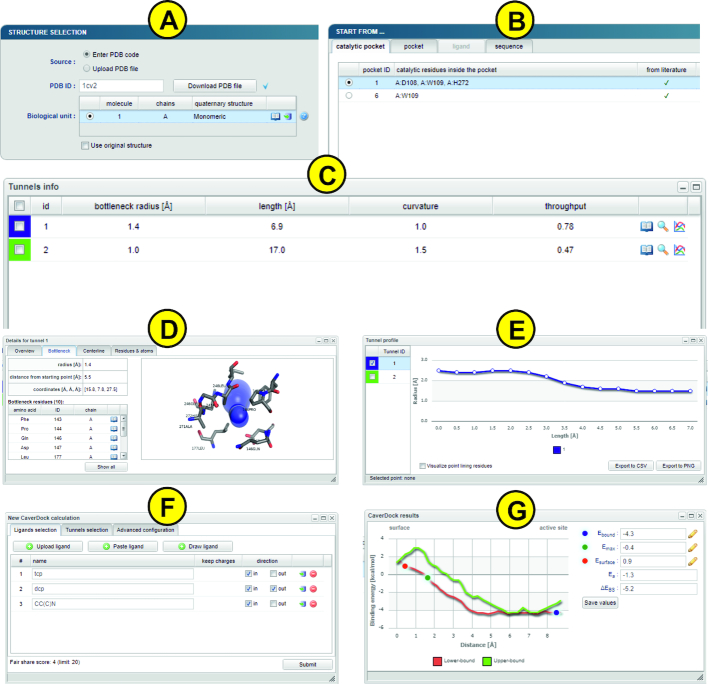
The graphical user interface of the Caver Web 1.0. The figure presents inputs and outputs obtained for the enzyme haloalkane dehalogenase LinB (PDB ID: 1CV2). (**A**) The ‘Select structure’ panel shows detected biological units for the provided protein structure. (**B**) The ‘Starting point’ panel for tunnel detection can be selected using four different methods. (**C**) The ‘Tunnel info’ panel provides an overview of the detected tunnels. (**D**) The ‘Tunnel details’ pop-up window presents detailed information about the selected tunnel. (**E**) The ‘Tunnel profile’ pop-up window shows the radius profile of the selected tunnels. (**F**) The ‘New CaverDock calculation’ pop-up window allows users to perform ligand transport analyses. (**G**) The ‘CaverDock results’ pop-up window displays calculated energy profiles for the selected ligand.

The only mandatory input is the tertiary protein structure, which can be either specified by the accession code to the Protein Data Bank database or uploaded as a file in the PDB or the CIF format (Figure 2A). Once the structure is loaded, the MakeMultimer tool is automatically executed to detect the biological units. More details about each unit and their image preview can be shown by clicking the ‘book’ icon available on each row. The generated PDB file containing the biological unit can also be downloaded using the ‘download’ icon.

The next step is the selection of the starting point for the tunnel detection (Figure 2B). The page integrates the JSmol (39) molecular viewer which provides a visualization support to all modes and allows an immediate and interactive check of the current starting point position (represented as a red ball). Currently, we support four modes, available via separated tabs. The ‘Catalytic pocket’ mode is suitable for enzymes and combines detected pockets with essential residues obtained from the Mechanism and Catalytic Site Atlas and the SwissProt databases. For each catalytic pocket, a list of assigned essential residues, pocket relevance score, volume and the estimated druggability score are available. Once a particular pocket is selected, all surrounding residues are visualized as sticks and the pocket shape is represented as an isosurface. The position of the starting point is calculated as the average centre of mass of all residues of the selected pocket. The second mode ‘Pocket’ allows users to start from any detected pocket making it useful in the case when there are no essential residues available in the databases. By default, only top ten pockets are shown and ordered by their relevance. The rest is available on demand. Each pocket is described by its relevance, volume, and estimated druggability score. Furthermore, users can view the residues surrounding the pocket in the protein sequence. The starting point position is then calculated in the same way as for the ‘Catalytic pocket’ mode. The third mode is ‘Ligand’ and provides the possibility to place the starting point to the centre of the mass of any bound ligand. Each ligand is described using the formula, the name and the residue number. All ligands are visualized in sticks and distinguished using the different colours. The ‘Sequence’ mode allows users to select residues manually either from the sequence or directly from the visualized structure. Each selected residue is automatically visualized as sticks. The starting point position is calculated as the average centre of mass of selected residues. The ‘Manual tuning’ can be activated in all four cases of the starting point selection to adjust the x, y and z coordinates. Once the starting point is selected, users can adjust the parameters of Caver calculation. The parameters were described in the Workflow section.

### Output of tunnel analysis

Users can specify a preferred job title for an easier orientation among submitted jobs. Notifications about the status of calculations can be sent to a provided email address. All jobs are stored and are accessible at any time. Once a job is submitted, tunnels are calculated using Caver tool and an analysis page is displayed. This page is divided into four major sections described below.

#### Job information

This section provides basic information about the job such as the identifier and the title. It also allows the user to directly download several files: (i) ‘PyMOL session’ downloads a pre-generated session file for the popular visualization software PyMOL. It contains the uploaded protein structure and all the detected tunnels offering the user to perform a detailed visual analysis or generate publication-quality images. (ii) ‘Results zip’ downloads an archive containing raw data generated by Caver during the calculation. The data can be used for advanced analyses or they can be directly imported to Caver Analyst (40). (iii) ‘Caver configuration’ opens a pop-up window with a complete configuration file used for the calculation. (iv) ‘Caver log’ opens a pop-up window with a raw textual output of Caver and provides details about the calculation process.

#### Tunnels info

The ‘Tunnels info’ section lists all identified tunnels and their selected properties (Figure 2C): (i) ‘bottleneck radius’ provides the maximal probe size which can fit in the narrowest part of the tunnel; (ii) ‘length’ quantifies the length of the tunnel from the starting point to the protein surface; (iii) ‘curvature’ describes the shape of the tunnel as the ratio between the length of the tunnel and the shortest possible distance between the starting point and the tunnel ending point; and (iv) ‘throughput’ reflects the probability that the pathway is used as a route for transport of the substances using the formula e^−cost^, where e is Euler's number and the cost is a function defined as:

\begin{equation*}\mathop \smallint \nolimits_0^L r{\left( l \right)^{ - 2}}\ dl\end{equation*}

where *L* is a length of path, *r*(*l*) is a function defining the radius of the largest ball which does not collide with the atoms of the structure and is centred at the point on the pathway axis in the distance *l* from the starting vertex (15). Every tunnel can be visualized by ticking the relevant checkbox and zoomed via the magnifying glass icon. Using the ‘book’ and the ‘chart’ icons, the ‘Tunnel details’ and the ‘Tunnel profile’ pop-up windows can be opened.

#### Tunnel details

The ‘Tunnel details’ pop-up window (Figure 2D) is organized into four tabs: (i) ‘Overview’ contains the important properties of the tunnel and a static picture containing the protein as a cartoon and the tunnel visualized by spheres; (ii) ‘Bottleneck’ contains details about the narrowest part of the tunnel (bottleneck) including a list of surrounding residues and a static picture of the bottleneck with the tunnel visualized as spheres and surrounding residues as sticks; (iii) ‘Centreline’ lists all centres of the spheres along the tunnel centreline with their distance from the starting vertex on the Voronoi diagram, radius, coordinates of the centre and the Euclidean distance from the starting point; (iv) ‘Residues & atoms’ contains the list of all residues surrounding the tunnel.

#### Tunnel profile

The ‘Tunnel profile’ pop-up window (Figure 2E) allows a comparative analysis of tunnel profiles, i.e., the tunnel radius over the distance along the tunnel centreline. Users can select one or more tunnels from the table on the left and the graphs are automatically generated. Moreover, every data point is interactive and allows a selection of the proper tunnel sphere in the visualization. The displayed graphs can be downloaded either as CSV files or PNG images.

#### Protein visualization

The protein and all the detected tunnels can be interactively visualized directly in the web browser using the JSmol applet. Users can choose to visualize the protein structures using several commonly used visualization styles, display a starting point and a starting pocket, show detected tunnels as balls or line, and visualize their neighbouring residues.

### Input of analysis of ligand transport

The last section of the output page from the tunnel calculation is devoted to an [optional] analysis of ligands transport through the tunnels.

#### Ligand transport analyses

The ‘Ligand transport analyses’ panel lists all CaverDock calculations with the basic information about the selected ligand, tunnel and the direction of the passage: (i) in – from the bulk solvent to the active site and (ii) out – from the active site to the bulk solvent. The status of each job is indicated as an icon - a green tick for successfully finished jobs, the ‘zzz’ icon for jobs waiting in a queue, an animated circle for currently running jobs and a red cross for failed jobs. More details about the job can be displayed by clicking on the ‘book’ icon. The log file containing all outputs generated during the calculation can be viewed using the ‘text file’ icon. Raw data can be directly downloaded using the ‘download’ icon. The ‘Export data’ button generates an Excel workbook with a summary sheet as well as a separate sheet for each job containing calculated energies (named by their identifier). A PDF report containing information about tunnels, jobs and energy plots can be generated by clicking on the ‘Generate report’ button.

#### Start new calculation

The ‘Start new calculation’ pop-up window (Figure 2F) is divided into three tabs: ‘Ligands selection’, ‘Tunnels selection’ and ‘Advanced configuration’. In the first tab, users have three ways of providing the only mandatory input: (i) ‘Upload ligand’ allows the user to upload the ligand in any format supported by the Open Babel (41); (ii) ‘Paste ligand’ supports the input either in SMILES format or as an accession code to ZINC15 database (42) and (iii) ‘Draw ligand’ provides the possibility to draw ligand's structure manually using the interactive molecular editor JSME (43). Users can specify a preferred name for each ligand and a desired direction ‘in’ or ‘out’ of the active site. Molecules uploaded in mol2 format can also keep their original charges. The second tab contains the list of all tunnels and allows the user to make their selection for the analysis. The last tab allows a modification of two parameters for the CaverDock calculation: ‘Discretization delta’ and ‘Calculation mode’ which were described in the Workflow section. Users can also select the ligands that should be kept in the structure during the analysis. The residue names considered during the tunnel detection are automatically selected by default. Since users can upload multiple ligands and select multiple tunnels, the submission can easily lead to a combinatorial explosion. To ensure fairness among users and prevent overloading of the computational resources, the number of concurrently running calculations is limited using a fair share score: *F* = *F*_C_ + (*L*_IN_ + *L*_OUT_) * *T* * *M*, where *F*_C_ is the fair share of currently running jobs, *L*_IN_ and *L*_OUT_ is the number of ligands passing in and out, respectively, *T* is the number of tunnels and *M* is the calculation mode coefficient (1 for lower-bound calculation, 1.5 for upper-bound calculation).

### Output of ligand transport analysis

#### Energy profile

The ‘Energy profile’ pop-up window (Figure 2G) shows the graph of the calculated binding energies for each disc. Furthermore, the window also enables an automatic calculation of the activating energy and the energy difference between ligand bound on the surface and in the active site. The users have to interactively select three points from the graph: (i) *E*_B_ – the energy minimum of the ligand bound in the active site; (ii) *E*_MAX_ – the maximum energy of the transition and (iii) *E*_S_ – the energy minimum of ligand bound in the tunnel mouth. The ‘Save values’ button stores the values in the report file.

#### Generate report

The ‘Generate report’ pop-up window is a configuration dialog allowing users to adapt the content and the format of the report. It is divided into two tabs. The first one contains the list of all successfully finished jobs allowing users to select which jobs should be included in the report. The second tab focused on energy profiles enables user selection of the scaling mode of all graph axes (trajectory, energy, and tunnel radius): (i) ‘Automatic’ scales the axis based on the minimal and maximal values of each job separately; (ii) ‘Automatic normalization’ scales the axis based on the minimal and maximal values for all selected jobs and (iii) ‘Manual limits’ scales the axis to the manually entered values.

### Use cases

The Caver Web tool can be used to address various biochemical problems. Three tutorials presented here and on the web portal provide an overview how Caver Web can be used: (i) to compare tunnels of different enzymes, (ii) to compare the passage of ligands via different tunnels of an enzyme and (iii) to screen a library of ligands for their passage through tunnels.

#### Case 1. Comparing the access tunnels of haloalkane dehalogenases

A comparison of protein tunnels can provide new insights into the structural elements coding for functional differences (2,11,44). Here, we studied the tunnels of five haloalkane dehalogenases (LinB, DmmA, DbjA, DhaA and DhlA), which catalyze the cleavage of carbon–halogen bonds in various halogenated hydrocarbons. These enzymes are closely related and their catalytic residues are conserved, yet their substrate preferences vary significantly (45,46). With the Caver Web tool we can show that the enzymes with more constricted tunnels (bottleneck < 1.5 Å) tend to be most effective with small substrates, e.g., DhlA with 1,2-dichloroethane and LinB with 1,2-dibromoethane. DmmA with the widest tunnels (bottleneck 2.5 Å) prefers the larger substrate 4-bromobutanenitrile. Conformational changes will be needed for binding of larger molecules to haloalkane dehalogenases via narrow tunnels (47).

#### Case 2. Studying paracetamol binding to the human cytochrome P450 3A4

Human cytochrome P450 enzymes (CYPs) metabolize a wide range of different substrates. The enzymes show a broad substrate specificity and possess multiple tunnels leading from the protein surface to the catalytic site. CYP3A4 is the main drug metabolizing enzyme in the liver, participating in the metabolism of ∼30% of available drugs (48,49). One of its substrates, paracetamol, is a common analgesic and antipyretic drug. Caver Web calculations revealed that the most preferred route for paracetamol binding to CYP3A4 is via the tunnel #2. Paracetamol can also bind through the tunnel #3, while its binding through the tunnels #1 and #4 requires conformational changes.

#### Case 3. Virtual screening of leukotriene A4 hydrolase/aminopeptidase inhibitors

Virtual screening is a well-established technique for drug design and there are many web services available for this purpose (50). Caver Web enables docking of ligands along a tunnel. This procedure significantly enhances the sampling region as compared to the classical docking. Our target the leukotriene A4 hydrolase/aminopeptidase (EC 3.3.2.6), is a bifunctional zinc metalloenzyme that catalyses the formation of the chemotactic agent LTB4, a key lipid mediator in the immune response. We screened 21 ligands and the resulting binding energy profiles were used for ligand ranking. We found out that the inhibitors ibuprofen and flurbiprofen have the easiest passage through the main tunnel. An additional finding was that oxaprozin binds stronger inside the tunnel than in the active site, which might indicate an inhibition mechanism based on a tunnel blockage. Such information would not be available from a classical virtual screening study targeting only the active site.

## CONCLUSIONS AND OUTLOOK

Caver Web 1.0 is a novel web server for structural and functional analysis of the tunnels and channels in protein structures. The tool complements tunnels and channels detection by an explicit analysis of ligand transport (Table 1). This unique functionality dramatically expands its use towards virtual screening in drug design applications. The server provides a simple and easy-to-use graphical user interface. Importantly, Caver Web integrates several automated helper procedures that guide the users through the workflow. They assist a correct setup of the calculation without a deep understanding of the setup and navigate the interpretation of the data obtained by the individual integrated tools. Caver Web improves the results of virtual screenings by analyzing the ability of potential inhibitors to reach their binding positions. The limitations of the web server relate to its simple interface. Some of the advanced analyses offered by the stand-alone versions of the software could be difficult to conduct via the web interface. Moreover, an analysis of extensive datasets, such as large libraries of ligands or protein assemblies from molecular dynamic trajectories, is also restricted due to the available computational resources.

**Table 1.** Comparison of Caver Web with available servers for detection of tunnels and channels in proteins and ligand transport analysis. Caver Web is currently the only tool which provides a one-stop shop for tunnel/channel identification and analysis of transport processes. Comprehensive comparison of Caver and CaverDock with other tools can be found in their primary publications (15,29).

		Tunnels and channels analysis		Ligand transport analysis			
Software	Input	Supported	Starting point selection	Supported	Ligand source	Output	Ref.
Caver Web	PDB ID^b^, PDB/CIF file^b^	Yes	Catalytic pocket, pocket, ligands, residues, coordinates	Yes	ZINC15, user file, drawing	Tunnels/channels, ligand trajectory, energy profile	this study
MolAxis	PDB ID, PDB file	Yes	Largest void, coordinates	No	-^d^	Tunnels/channels	(16)
MoleOnline	PDB ID^c^, CIF/PDB file^c^	Yes	Catalytic residues, residues, coordinates, pocket, pattern	No	-^d^	Tunnels/channels	(51)
BetaCavityWeb	PDB ID, PDB file	Yes	*Not required*	No	-^d^	Tunnels/channels	(52)
PoreWalker	PDB file	Yes	*Not required*	No	-^d^	Channels	(12)
ChExVis	PDB ID, PDB file	Yes	Catalytic residues, HETATM records, residues	No	-^d^	Tunnels/channels	(53)
MoMA-LigPath^a^	PDB file	No	-^d^	Yes	Part of PDB file	Ligand trajectory	(32)

^a^Web server SLITHER for ligand transport analysis was not accessible in the time of writing.

^b^Biological units detection by MakeMultimer.

^c^Biological units fetched from the PDBe database (54).

^d^Not applicable.

New features will be implemented in the future versions of Caver Web. Firstly, we plan to optimize the position of the starting point within the pockets. The current algorithm places the point in the middle of the pocket, which in some cases leads to a shortening of the tunnel length. Therefore, we will develop a new algorithm, which will automatically push the starting point deeper into the pocket. Secondly, we will focus on protein dynamics, which can be crucial for efficient ligand transport through access tunnels in many biological systems. An incorporation of the side chains’ flexibility or an analysis of molecular ensembles can provide important insights into the tunnel dynamics and their importance for transport processes. Thirdly, a possibility to introduce mutations to tunnel-lining or bottleneck residues and then to recalculate analyses will expand in protein engineering. Finally, the currently used visualization tool JSmol will be replaced by the Mol* tool, which is being developed by PDBe and RCSB PDB teams.

## Supplementary Material

gkz378_Supplemental_Files
