# Supplementary material for: Caver Web 1.0: identification of tunnels and channels in proteins and analysis of ligand transport
Source: Nucleic Acids Res. 2019 May 22;47(W1):W414–22. doi: 10.1093/nar/gkz378 (PMC6602463; doi:10.1093/nar/gkz378)
Supplement: gkz378_Supplemental_Files [file gkz378_supplemental_files.zip › Stourac_NAR_Web_Case1.docx]

## Case 1: Comparing the Access Tunnels of the Haloalkane Dehalogenases

Haloalkane dehalogenases catalyze the cleavage of carbon-halogen bonds of many types of halogenated hydrocarbons. These enzymes are useful biocatalysts for the purification of industrial waste waters, decontamination of warfare chemicals and as biorecognition elements in biosensors. Haloalkane dehalogenases are closely related and their catalytic residues are nearly identical, but their substrate preferences vary (1, 2). In this tutorial, we will compare the access tunnels of five haloalkane dehalogenases using the Caver Web to shed light on the differences in their substrate specificities (Table 1).

**Table 1.** The studied haloalkane dehalogenases and their Protein Data Bank identifiers (PDB IDs).

| Haloalkane dehalogenase | PDB ID |
| --- | --- |
| DmmA | 3U1T |
| DhaA | 4HZG |
| DhlA | 2YXP |
| DbjA | 3A2M |
| LinB | 1MJ5 |

This tutorial consists of following steps:

1. Selecting the protein structure
2. Determining the tunnel starting point
3. Setting up the Caver parameters
4. Analyzing the tunnels
5. Collecting the data

### Selecting the protein structure

To use a protein structure from the PDB, you enter its PDB id to the field on the Caver Web front page and click on “Download PDB file” (Figure 1). Alternatively, you can also upload your own .pdb file. Insert 3U1T to the PDB ID field and press “Download PDB file” button.


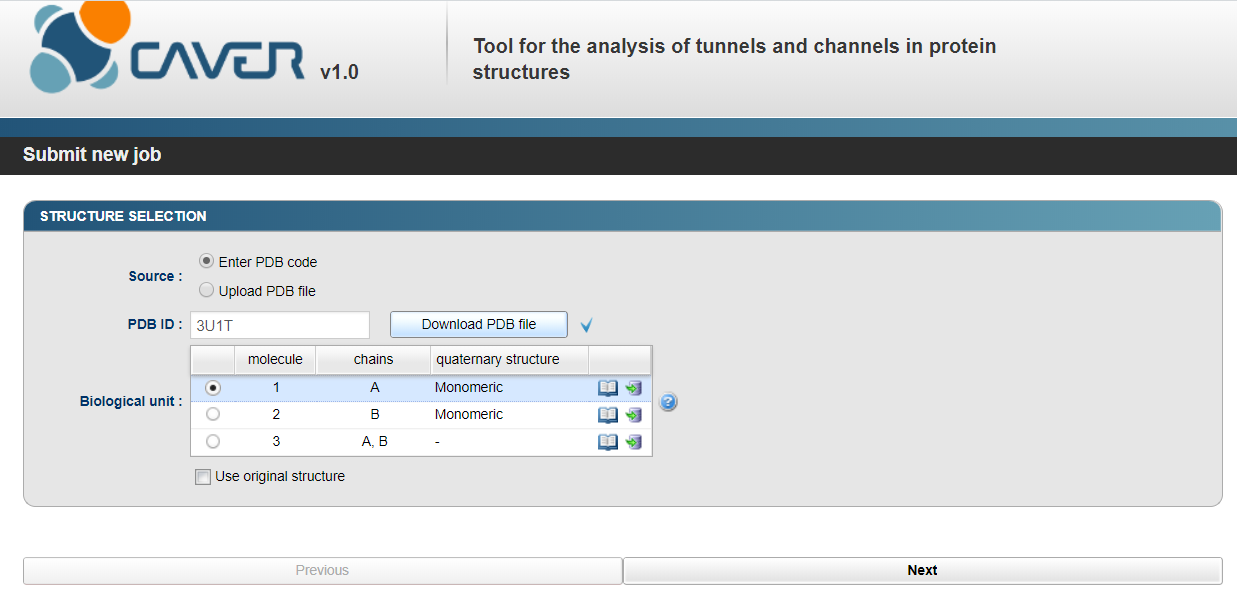


**Figure 1.** The Structure selection page.

Next, you need to select the biological unit. You can see the details of the biological units by clicking on the book icon at the end of the row. In this tutorial, we will use chain A. Select chain A by clicking its row. Click “Next” will bring you to the selection of tunnel starting point.

### Determining the tunnel starting point

Caver Web offers multiple options for selecting the tunnel starting point, which is typically located inside the active site. You can select it from the catalytic site information extracted from UniProt, from the calculated pockets, using the ligands present in the pdb structure or selecting residues from the sequence. Caver Web also provides visualization of the protein structure to aid the selection of the starting point. When entering the tunnel starting point phase, Caver Web will automatically detects the pockets which might take a while if your protein is large (>500 residues).

In this tutorial we will use the pocket option (Figure 2). Click on “pocket” tab to see the details of the found pockets. You can see the statistics of the pockets, including volume and a druggability score in the table. The druggability score tells on scale from 0 to 1 how much the pocket resembles the small molecule binding pockets. Select pocket #1 by clicking on the dot at the start of its row.

You can click on the “Show in sequence” button below the pocket overview window to see the placement of those residues in the protein sequence (Figure 3). In the visualization window, you will see the selected pocket, the surrounding residues as ball-and-stick presentation and a red ball indicating the starting point for tunnel calculation (Figure 4). You will see the exact coordinates printed on the bottom of the page. Click “Next” once you are done with visualizing your structure and the starting point.

**
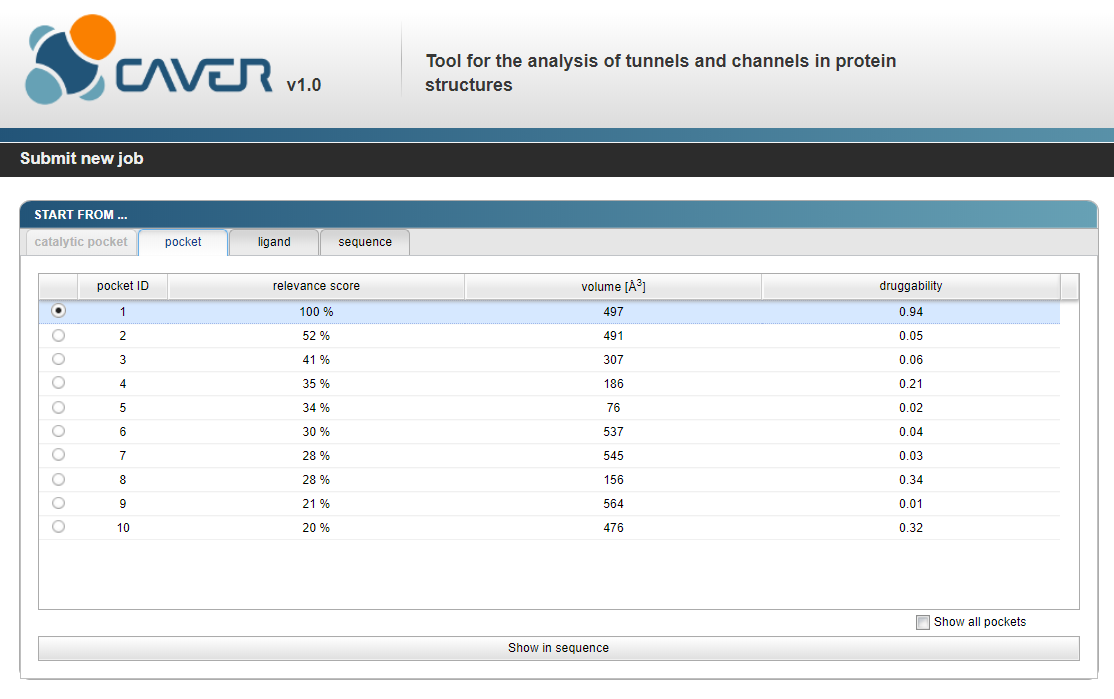
**

**Figure 2.** The pocket overview window.

**
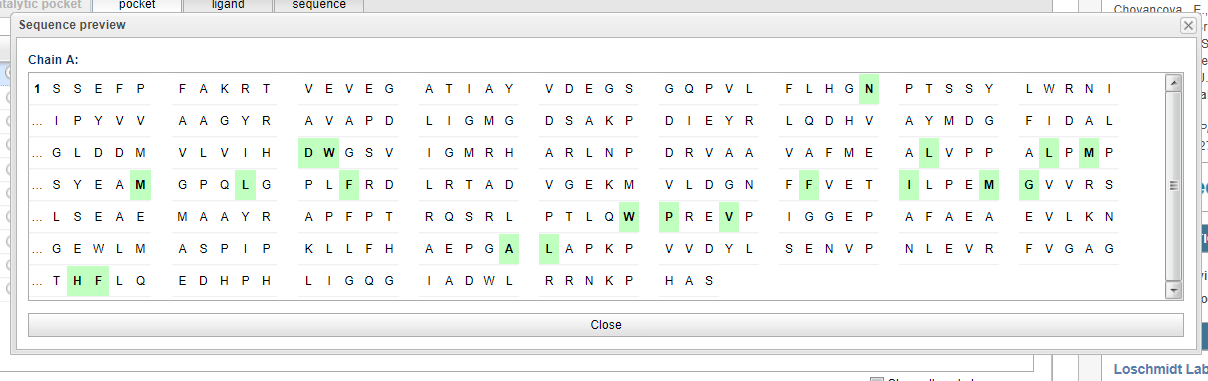
**

**Figure 3.** The sequence view of the residues surrounding the selected pocket.


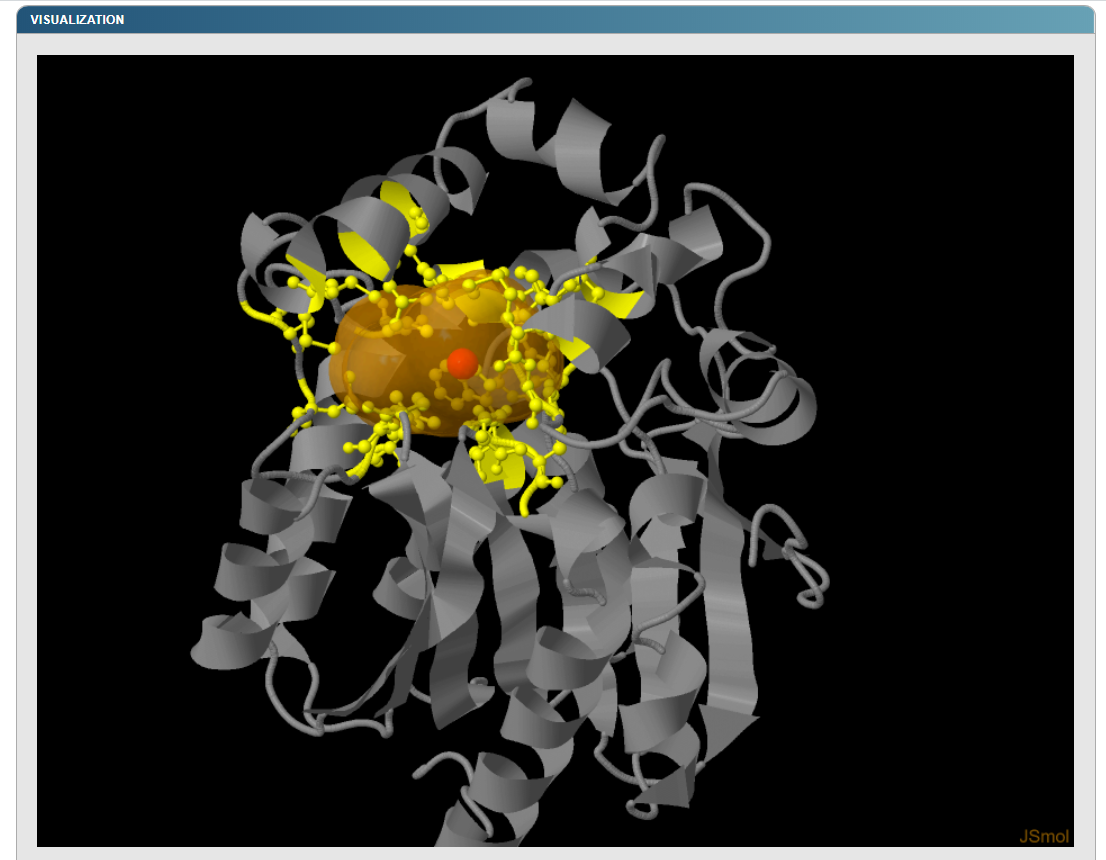


**Figure 4.** The visualization of the selected pocket.

### Setting up the Caver parameters

The Caver parameters can be set on the next page. These parameters determine how the tunnels are calculated (Figure 5). You can select parts of the structure to be included or excluded during the tunnel calculation. For example, sometimes cofactor molecule in the active site can be included in the calculation. You can include a name for your job and an email address to get notified once the calculations are finished. A typical job of tunnels calculation takes only a few minutes.

You will find more information about the individual settings by clicking the “?” icon next to them. There are also buttons to reset the values to defaults. The default settings have been determined through extensive testing and they should provide users with reliable data in most cases. Standard settings should be used throughout this tutorial. If you changed some values, click on “Reset values” and then click on “Submit job”.

**
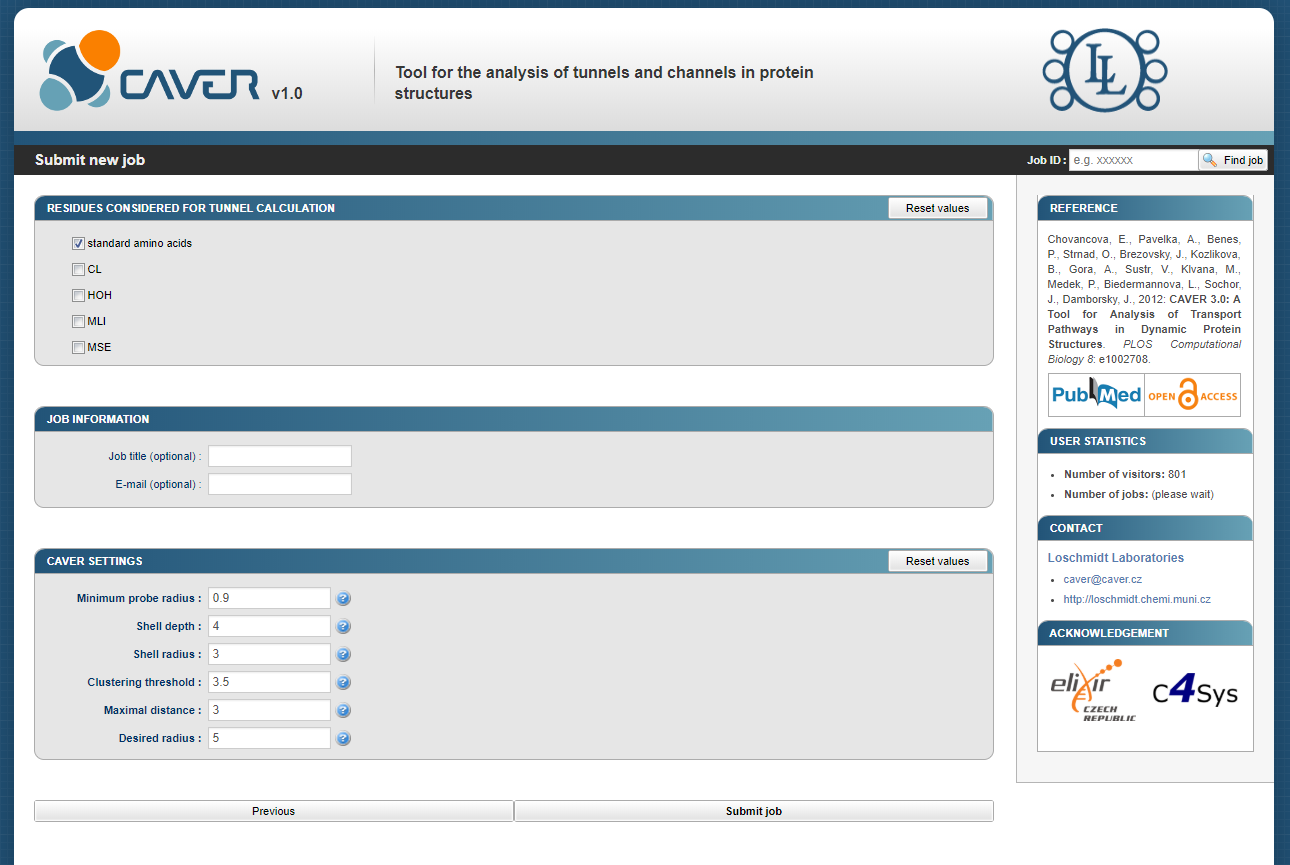
**

**Figure 5.** Settings for the tunnel calculation parameters.

### Analyzing the tunnels

You will have an overview of the tunnel statistics in the window “Tunnels info”. If no tunnels are found, you could try using a smaller probe size (Minimum probe radius) on the previous page. The Tunnels info panel shows the general statistics of the tunnels, such as the bottleneck radius and the length of the tunnel (Figure 6). The small icons at the end of each tunnel let you take a look at the tunnel details, zoom in on the tunnel in the visualization and to see the tunnel profile – plotted radius *versus* length. You can download the visualization of the tunnels as a PyMOL session, all the results together with the configuration and log files of the Caver run as a ZIP file.

The visualization settings allow different visualization styles for the protein, the tunnels and the pocket. You can visualize the tunnel as spheres or tracing its center line, and the protein can be presented as cartoon, sticks, wireframe, balls and sticks, trace and backbone. You can also visualize the pocket with the starting point for the tunnel calculation. It is possible to export images directly from the Caver Web interface, just click the “Save image button” and you can download the visualized image as a PNG file.

**
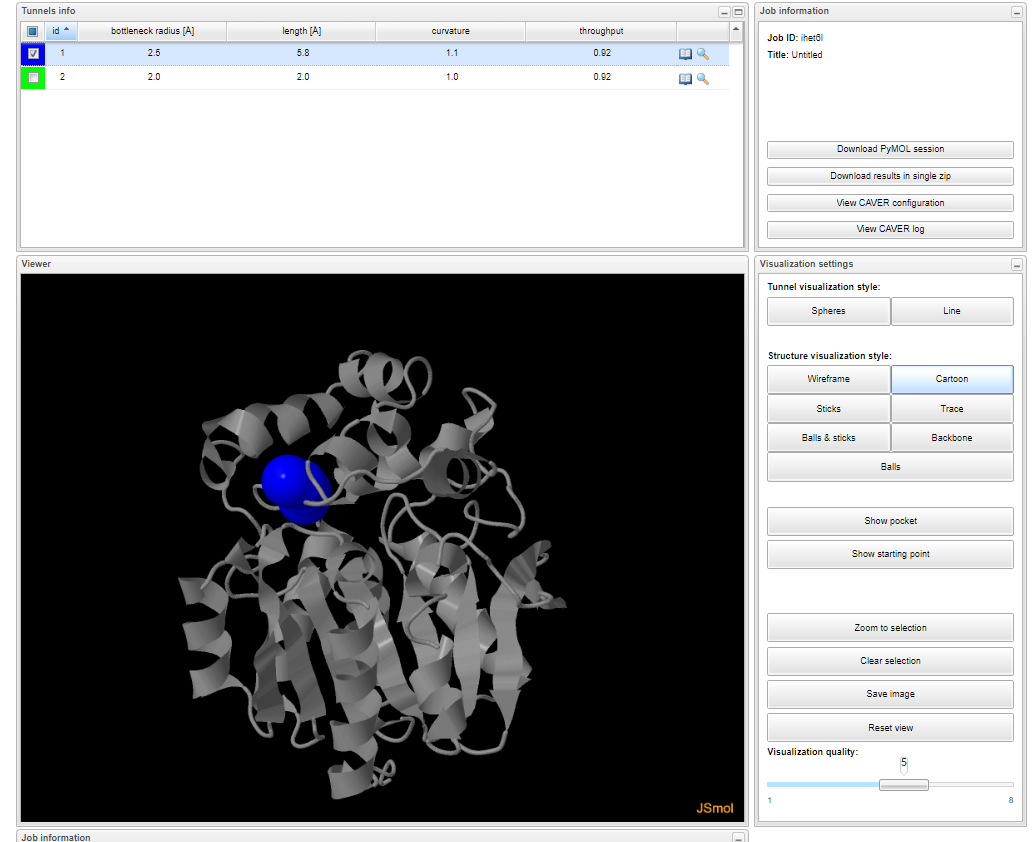
**

**Figure 6.** The results page of tunnel calculation.

If you want to take a closer look to the details of a single tunnel, click on the “book” icon on the tunnels row in the tunnel “Overview” table. It will open a new window with tabs Overview, Bottlenecks, Centerline and Residues & Atoms (Figure 7). The overview shows details of the tunnel statistics with a visualization of the tunnel profile. In the “Bottleneck” tab you will see the tightest point of the tunnel with the surrounding residues. The “Centerline” and “Residues & atoms” tabs will show you the coordinates for the tunnel centerline and a detailed list of the residues that surround the tunnel.


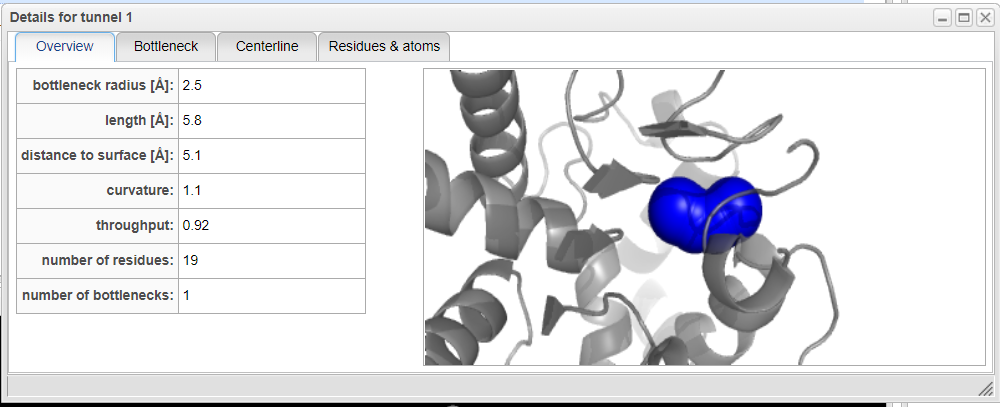


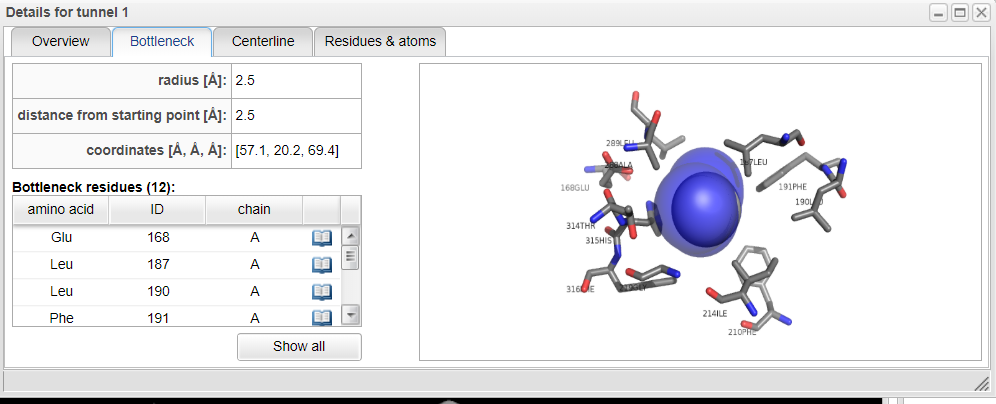


**Figure 7.** The tunnel details window.

### Collecting the data

You will need to repeat the steps 1-4 with the four remaining dehalogenases and collect the data from each of the runs to reproduce the data in this tutorial. Note that the active site pocket of the DhlA (2YXP) corresponds to #2 instead of #1. Moreover, DhlA has very narrow tunnels and the probe size needs to be reduced to 0.7 Å since all the tunnels have smaller bottleneck than 0.9 Å. Lowering the bottleneck radius would not affect the results with other structures, but you would see a larger number of identified tunnels in the “Tunnel overview” table.

The details of the tunnel #1 of all the studied haloalkane dehalogenases have been collected in the Table 2. You can see that the bottleneck radius varies from 0.8 Å of DhlA to 2.5 Å of DmmA. The curvature of the tunnels remains similar, indicating that the catalytic sites of haloalkane dehalogenases connect to the surrounding solvent with relatively straight tunnels. The throughput is calculated from the bottleneck radius and the curvature, with values closer to 1 indicating an easier-to-pass tunnel.

**Table 2.** The tunnel characteristics of the haloalkane dehalogenases from the tunnel overview tables.

| Haloalkane dehalogenase | Bottleneck radius (Å) | Length  (Å) | Curvature | Throughput |
| --- | --- | --- | --- | --- |
| DmmA | 2.5 | 5.8 | 1.1 | 0.92 |
| DhaA | 1.6 | 9.2 | 1.2 | 0.74 |
| DhlA | 0.8 | 13.2 | 1.2 | 0.48 |
| DbjA | 2.3 | 2.8 | 1.1 | 0.92 |
| LinB | 1.3 | 8.3 | 1.2 | 0.71 |

The length of the tunnels varies from 2.8 Å to 13.2 Å. This is due to using the pocket center as the calculation starting point. DbjA and DmmA with the shortest tunnels have large, open and easily accessible catalytic sites which makes the pockets span to the surface, thus bringing the center of the pockets close to the surface and shortening the length of the tunnels. Depending on the biological question addressed by the calculation, one can remake the analysis with a starting point defined by the catalytic residues and obtain different results for the tunnel length.

The haloalkane dehalogenases possess a broad substrate specificity and some of them show activity towards at least one of the poorly degradable toxic pollutants such as 1,2-dichloroethane, 1,2-dichloropropane or 1,2,3-trichloropropane. The enzymes with the narrower tunnels prefer smaller substrates and convert them with the higher catalytic efficiency. The preferred cognate substrate for DhlA with the narrowest tunnel is 1,2-dichloroethane, a small and hydrophilic compound. The preferred substrate for LinB is 1,2-dibromoethane, again a small and hydrophilic compound. DmmA with the widest tunnel prefers a longer molecule 4-bromobutanenitrile. Curiously, also the enzymes with the narrowest tunnels, LinB and DhlA, can accommodate bigger substrates thanks to conformational changes during the ligand binding (3), which will be addressed in the future versions of the Caver Web tool.

### Conclusions

Significant differences in the anatomies and physicochemical properties of the access tunnels of the haloalkane dehalogenases were observed in this tutorial. The enzymes with the narrower access tunnels (DhlA and LinB) prefer smaller substrates such as 1,2-dichloroethane and 1,2-dibromoethane, while the enzymes with wider openings (DmmA and DbjA) accepts larger substrates and show generally broader substrate specificity. The access tunnels represent the important structural features determining the catalytic properties of enzymes with buried active sites. The bulkier substrates require a conformational change of the enzyme to bind through the narrow tunnels. Modelling of these conformational changes will be implemented in the future versions of the Caver Web.

### References

1. Koudelakova,T., Chovancova,E., Brezovsky,J., Monincova,M., Fortova,A., Jarkovsky,J. and Damborsky,J. (2011) Substrate specificity of haloalkane dehalogenases. *Biochem. J.*, **435**, 345–354.

2. Gehret,J.J., Gu,L., Geders,T.W., Brown,W.C., Gerwick,L., Gerwick,W.H., Sherman,D.H. and Smith,J.L. (2012) Structure and activity of DmmA, a marine haloalkane dehalogenase. *Prot. Sci.*, **21**, 239–248.

3. Kokkonen,P., Bednar,D., Dockalova,V., Prokop,Z. and Damborsky,J. (2018) Conformational changes allow processing of bulky substrates by a haloalkane dehalogenase with a small and buried active site. *J. Biol. Chem.*, **293**, 11505–11512.
