# Supplementary material for: Caver Web 1.0: identification of tunnels and channels in proteins and analysis of ligand transport
Source: Nucleic Acids Res. 2019 May 22;47(W1):W414–22. doi: 10.1093/nar/gkz378 (PMC6602463; doi:10.1093/nar/gkz378)
Supplement: gkz378_Supplemental_Files [file gkz378_supplemental_files.zip › Stourac_NAR_Web_Case2_clean.docx]

## Case 2: Studying Paracetamol Binding to Human Cytochrome P450 3A4

CYP3A4 is the main drug metabolizing enzyme in the liver, participating in the metabolism of about 30% of available drugs (1, 2). There are multiple tunnels leading to the active site of this enzyme. The catalytic site of CYP3A4 contains a heme molecule, which is crucial for its monooxygenation reaction. The process of binding of paracetamol (acetaminophen) to the catalytic site of human cytochrome P450 CYP3A4 via different tunnels with the aim to identify preferred route will be studied in this tutorial.

The tutorial consists of following steps:

1. Calculating the tunnels
2. Uploading the ligand
3. Selecting the tunnels
4. Setting up parameters
5. Analyzing the results

### Calculating the tunnels

Load the crystal structure of human CYP3A4 (PDB ID 4NY4) to Caver Web and calculate the tunnels based on the largest pocket found using default settings as described in the Tutorial 1. Do not accept the suggestion of a better starting position. This would lead to the tunnels being calculated on opposite side of the Heme group. Before submitting the tunnel calculation job, the Heme group [HEM] should be checked so it is considered for the tunnel calculation. In this tutorial we will focus on the four most significant tunnels shown in the Figure 1.


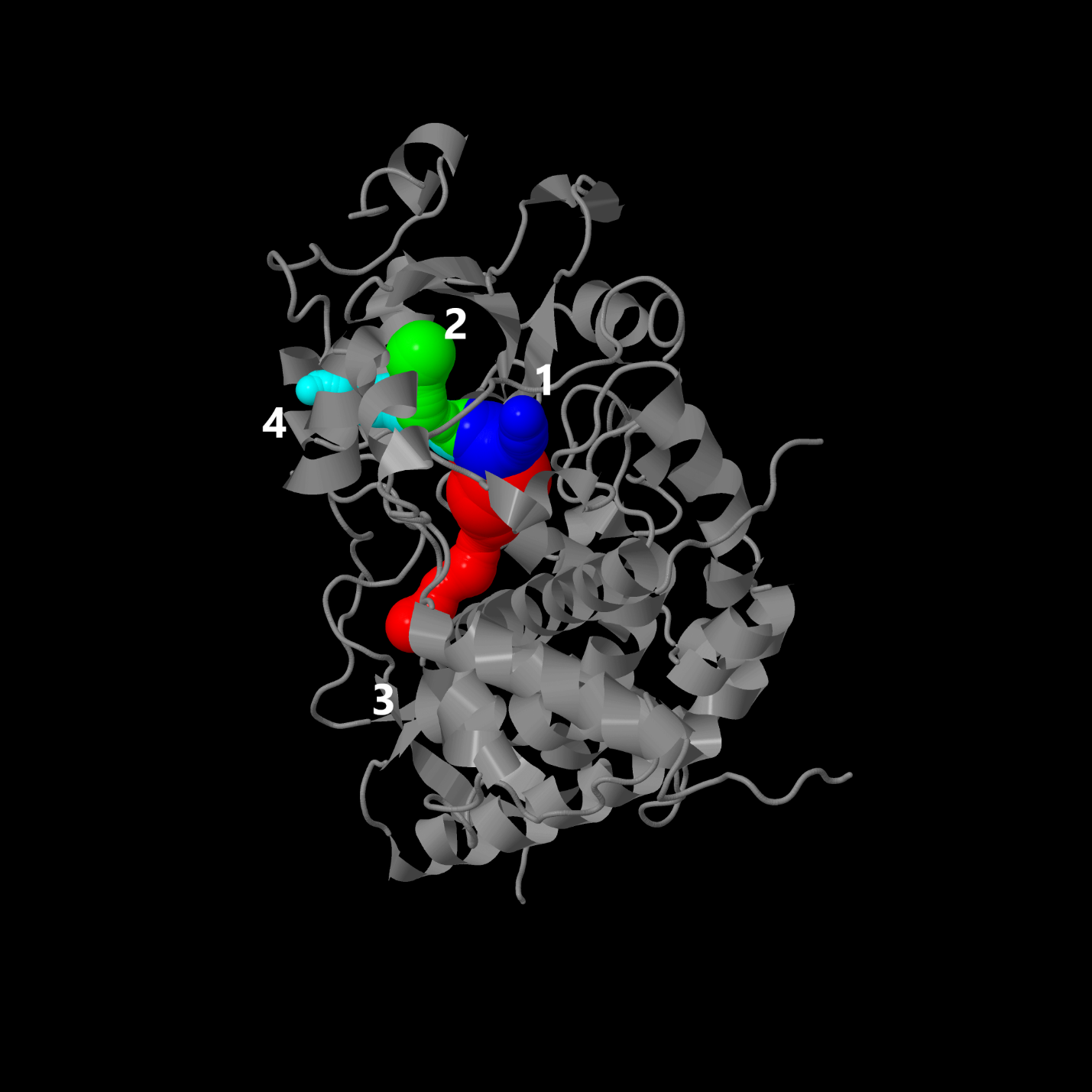


**Figure 1.** The most significant tunnels of human CYP3A4.

### Uploading the ligand

The CaverDock calculations are started from “Start new calculation” button, which is located under the tunnel visualization window on the tunnel analysis page. Pressing this button opens a window where you can set up a CaverDock calculation for one or more ligands through one or more tunnels (Figure 2).


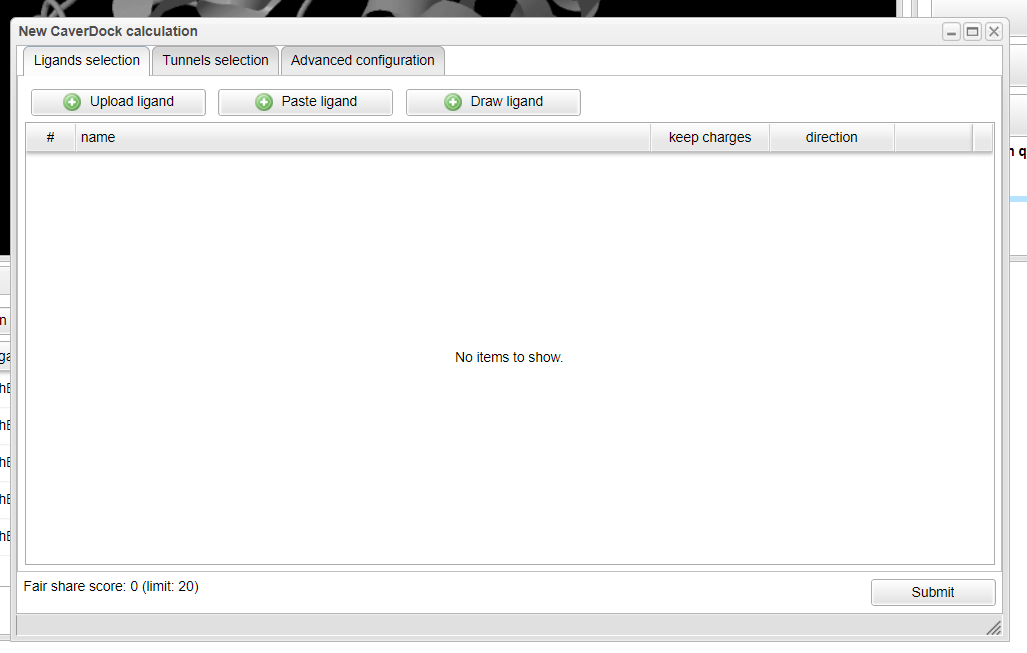


**Figure 2.** The CaverDock setup window.

To input the ligands, you can either upload your own files (.mol, .mol2 or .pdb), paste a ligand’s SMILES code or ZINC accession code, or draw the structure of the molecule in the ligand editor. The ZINC code for paracetamol is ZINC13550868. Input this code into the “Paste Ligand” tab and click on Process. You will see your ligand numbered as #1 on the Ligand selection window. The ligand’s name is acetaminophen, which is the synonym of paracetamol. You can rename it to paracetamol. On the row of the ligand are buttons to download the ligand structure and to delete the ligand from analysis. You can also select the directions of the calculation: (i) “in” for the transport of the ligand from protein surface to the active site cavity or (ii) “out” for the transport of the ligand from the active site cavity to the protein surface. Select the “in” direction to study the process of binding.

### Selecting the tunnels

Clicking on the “Tunnels selection” opens a tab similar to the tunnel overview, where you see the data of the tunnels (Figure 3). Select the tunnels 1, 2, 3 and 4.


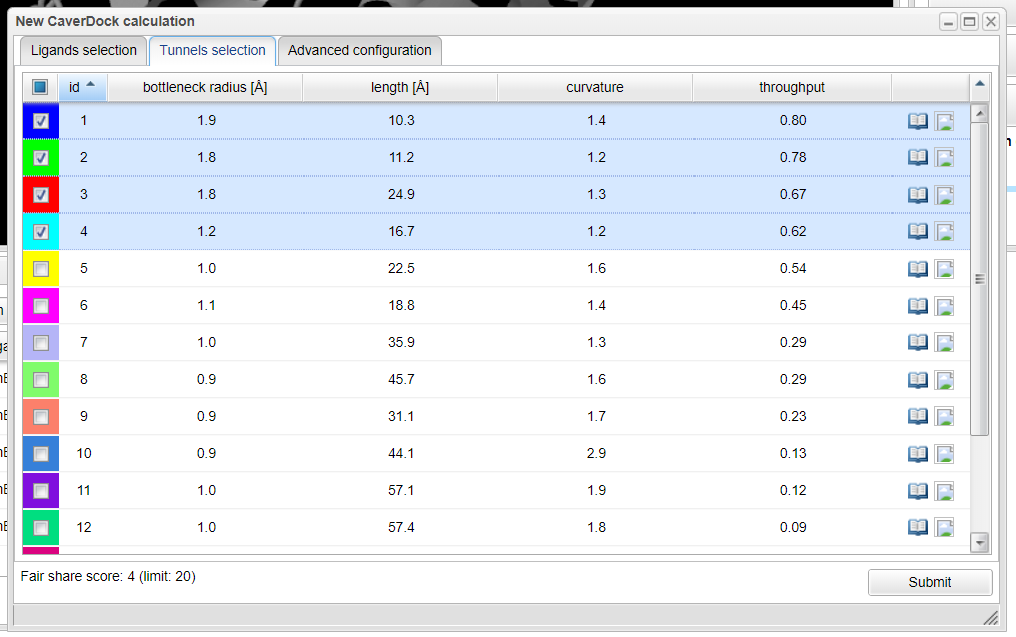


**Figure 3.** The tunnel selection tab.

### Setting up parameters

The tunnel discretization delta can be selected on the “Advanced configuration” tab. This parameter determines the resolution of the tunnel discretization into the disks. Lowering the value increases calculation time due to more docking runs. Increasing the value will speed up the calculation which might be beneficial if you have long tunnels and need to decrease the calculation time. In this tutorial we use the default parameters.

The calculation mode lets the user to choose to calculate the “upper-bound trajectory” in addition to “lower-bound trajectory”, which is calculated always. The upper-bound trajectory is a continuous trajectory where the ligand is not allowed to flip while moving through the tunnel. The calculation of the upper-bound trajectory will take significantly longer due to the heuristics and backtracking required for the continuity of the trajectory. We will only calculate the lower bound trajectory to save time in this tutorial.

Click on submit to submit your job. You will notice 4 jobs appearing on the CaverDock panel. The calculations take about 30 minutes. You can also access the previously calculated results using the ‘pre-calculated results’ link in the Example section on the web. Insert to code to the field at top right corner of the page.

### Analysis of results

You can visualize the energy profiles of the binding trajectories individually by clicking on the book icon on the finished jobs (Figure 4). You can select the points on the graph corresponding to the bound state (E_bound_), maximum energy barrier (E_max_) and unbound state (E_surface_). Click on the pencil icon next to the corresponding fields and click on the corresponding spot on the binding trajectory graph. In this case, the binding site will be on the far right and the surface on the far left. Caver Web will then automatically calculate an estimate of the binding energy barrier (E_A_) and the difference in energy of the bound and unbound states (E_BS_). Once you have saved the values, you can close the window. Find the energy values for all four tunnels and remember to press “Save values” for each of them.

**
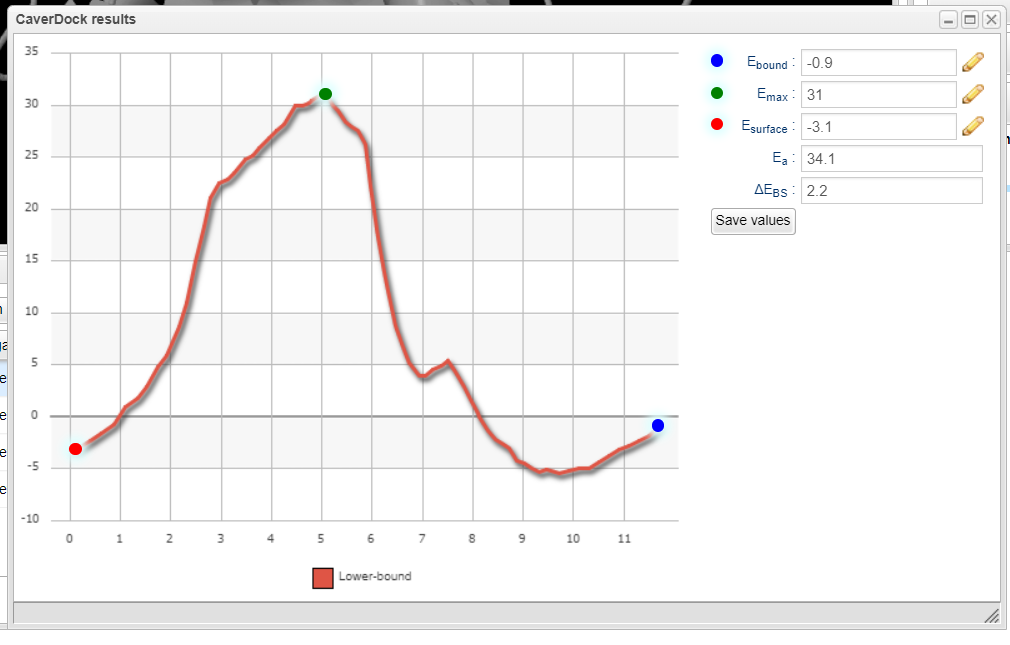
**

**Figure 4.** The binding trajectory of paracetamol going through the tunnel #1 in CYP3A4.

You can generate a CaverDock report where your results will be presented in a simple and readable PDF document. The report will be automatically created to allow comparison of the ligands/tunnels with each other by scaling the axes of the energy graphs. Press on the “Generate report” button below the binding trajectory table to open the “Generate CaverDock report” window (Figure 5). Select all ligands. You can see the settings on the “Energy profile” tab, where you can manually set your desired values for the graphs. We leave the settings to default in this tutorial. Press “Generate report” and save the resulting PDF file on your computer.


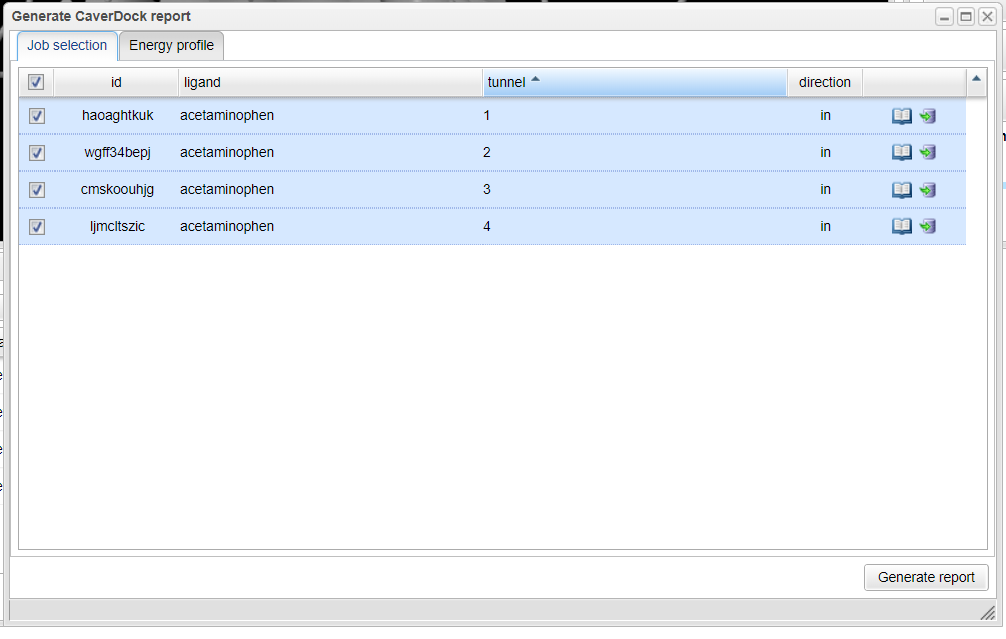


**Figure 5.** The window where you can create a CaverDock report.

The generated report is available <https://loschmidt.chemi.muni.cz/caverweb/data/case2_report.pdf>. It consists of the data from the Caver and the CaverDock calculation represented as tables on the first page, and the energy profiles on the second page. We can see from the tunnel statistics that the first four tunnels of CYP3A4 do not have large differences in their bottleneck radius, but their length varies significantly. It is impossible to guess which tunnel would be the preferred for paracetamol.

In the CaverDock results table, the energies for the bound state (E_bound_) are similar with the exception of tunnel #1. You can see in the energy profile of tunnel 1 that the binding energy starts to rise after a minimum at the active site from around 10 Å into the trajectory. Sometimes the CaverDock calculations try to dock the ligand too “deep” into the active site, where it clashes with the active site residues and causes unnatural binding energies. E_bound_ -5.6 kcal/mol could be found around 10 Å and should be used for further estimation of the energies.

Also, the surface energies should be similar (E_surface_). In this case, the difference come to tunnel #4, which has a tight bottleneck (1.5 Å) close to the surface. The bottleneck causes steric clashes in the docking and results in a high energetic barrier. In reality, the binding through this tunnel would induce conformational changes to accommodate the ligand.

It is unlikely that paracetamol binds through tunnels #1 or #4, based on the high energy barriers of binding (E_max_). Since tunnel #2 is shorter and its trajectory displays a smaller activation barrier (E_A_) than tunnel #3, it is the most probable binding route for paracetamol.

### Conclusions

The most preferred route for paracetamol binding to CYP3A4 is the tunnel #2. Paracetamol can also bind through the tunnel #3, while the binding through the tunnels #1 and #4 requires conformational changes. We note that the tunnel #2 corresponds to the tunnel #2a of the previous study, where opening was observed in molecular dynamics simulations (2).

### References

1. Haddad,A., Davis,M. and Lagman,R. (2007) The pharmacological importance of cytochrome CYP3A4 in the palliation of symptoms: review and recommendations for avoiding adverse drug interactions. *Support. Care Canc.*, **15**, 251–257.

2. Baylon,J.L., Lenov,I.L., Sligar,S.G. and Tajkhorshid,E. (2013) Characterizing the membrane-bound state of cytochrome P450 3A4: Structure, depth of insertion, and orientation. *J. Am. Chem. Soc.*, **135**, 8542–8551.
