# Supplementary material for: Caver Web 1.0: identification of tunnels and channels in proteins and analysis of ligand transport
Source: Nucleic Acids Res. 2019 May 22;47(W1):W414–22. doi: 10.1093/nar/gkz378 (PMC6602463; doi:10.1093/nar/gkz378)
Supplement: gkz378_Supplemental_Files [file gkz378_supplemental_files.zip › Stourac_NAR_Web_Case3_clean.docx]

## Case 3: Virtual Screening of Leukotriene A4 Hydrolase/Aminopeptidase Inhibitors

Virtual screening is a well-established technique for drug design and is available via several web services (1). Caver Web enables docking of the ligands along the access tunnels or channels and enhances the sampling region. The leukotriene A4 hydrolase/aminopeptidase (EC 3.3.2.6) is a bifunctional zinc metalloenzyme that catalyzes the formation of the chemotactic agent LTB4, which is a key lipid mediator and plays an important role in the immune response (2, 3). Binding trajectories of 21 anti-inflammatory drugs through the tunnel of the leukotriene A4 hydrolase/aminopeptidase will be analyzed in this tutorial.

This tutorial consists of following steps:

1. Protein structure selection
2. Determining the tunnel starting point
3. Setting up the CAVER parameters
4. Analyzing the tunnels
5. Selecting the ligands
6. Analyzing the data
7. Conclusions

### Protein structure selection

To use a protein structure from the PDB database, you enter its PDB id to the field on the Caver Web front page and click on “Download PDB file” (Figure 1). You can also upload your own .pdb-file. For this case study please insert 4L2L (3) into the PDB ID field and press “Download PDB file” button.

**
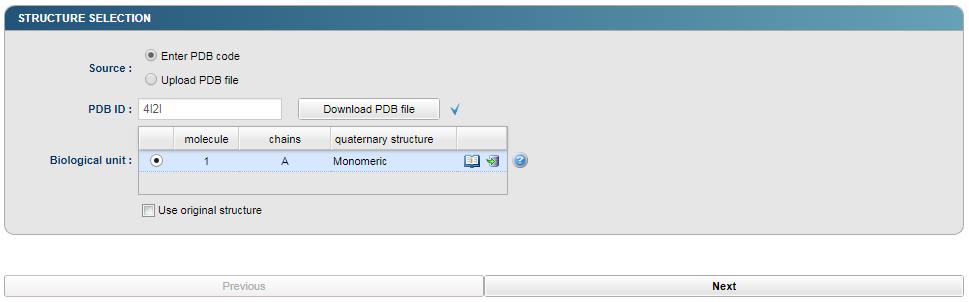
**

**Figure** **1.** The Structure selection page.

The protein analysed in this case study has only one biological unit. By clicking Next, the user will come to the following page, where the starting point may be defined.

### Determining the tunnel starting point

Caver Web offers several options for selecting the tunnel starting point. You can select one of the following options:

- key catalytic residues extracted from UniProt
- calculated pockets
- ligands present in the structure
- selecting residues from the sequence
- manual insertion of XYZ coordinates of starting point

Caver Web also provides visualization of the protein structure to aid the selection of the starting point. In this case study we will use the pocket option (Figure 2). Click on “Pocket” tab to see the details of the found pockets. You can see the statistics of the pockets, including volume and a druggability score in the table. The druggability score tells on scale from 0 to 1 how much the pocket resembles druggable (small molecule binding) pockets. Since we want to do a small virtual screening with known anti-inflammatory drugs, we want to select the pocket with the highest value for druggability. Select pocket ID 3 by clicking on the dot at the start of the row.

**
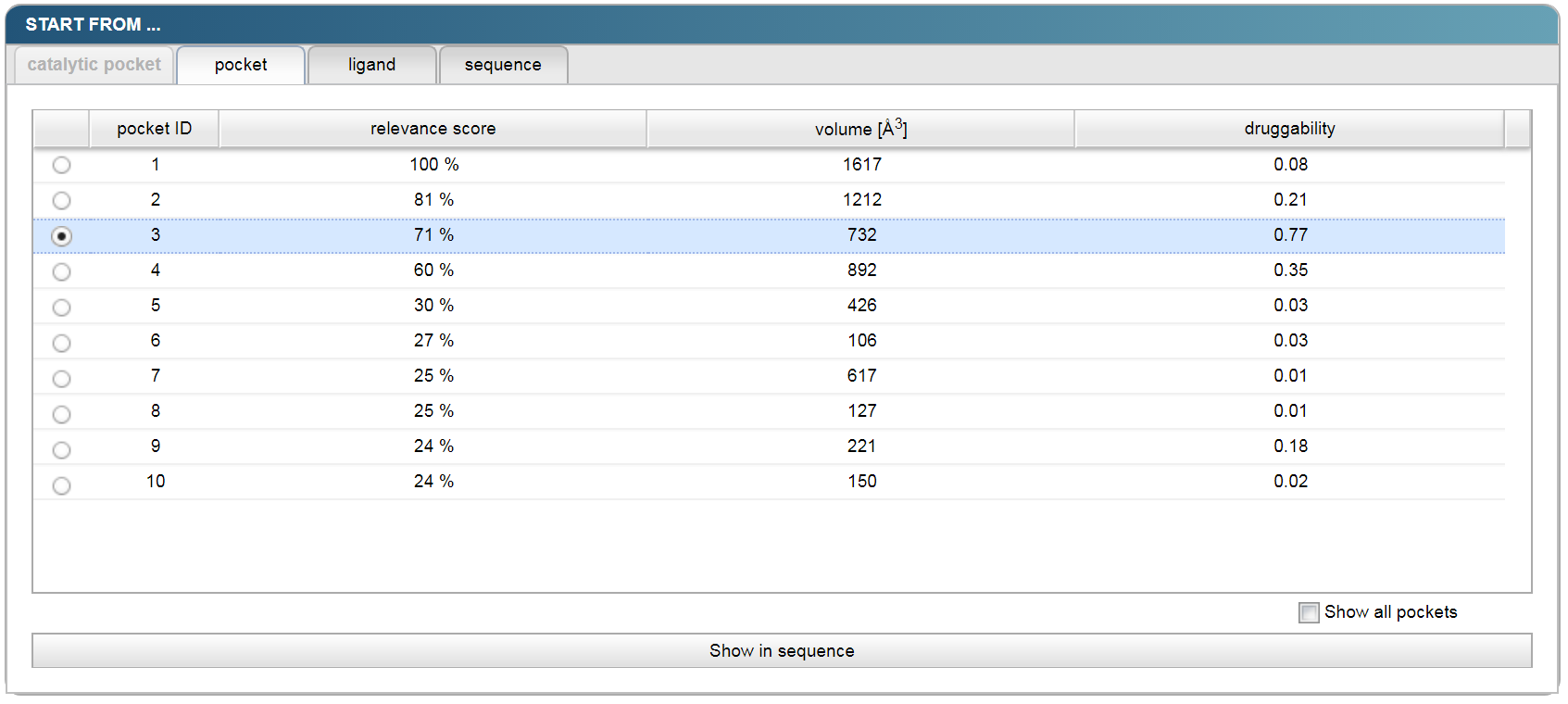
**

**Figure 2.** The Starting point selection window presenting properties of identified pockets.

When clicking the button “Show in sequence” below the pocket window, a new window will pop up. This window has the FASTA sequence of the target protein and shows, highlighted in green, which residues are on the chosen pocket (Figure 3).


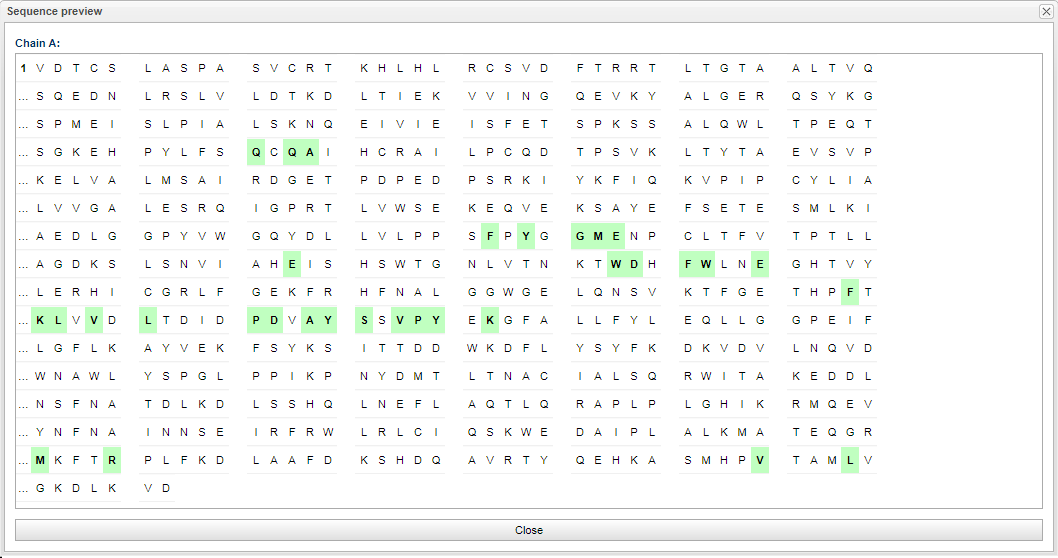


**Figure 3.** The sequence view of the residues surrounding the selected pocket.

In the visualization window, which is below the “Start From…” window, the selected pocket is shown as see through orange surface, the surrounding residues as yellow ball-and-stick representation and a red ball indicating the starting point for tunnel calculation. Below the protein and the chosen pocket visualization window, the user will see the xyz coordinates printed separately (Figure 4).

There is an option on the bottom right corner that allows the user to fine tune the starting point for the Caver calculation. For this case study however, the user should not fine tune the starting point. Once you are done visualizing, click “Next”. If you get a notification of a “possibly more reliable starting point found”, press Cancel. This way you will go to the next step with the pocket of your choosing.


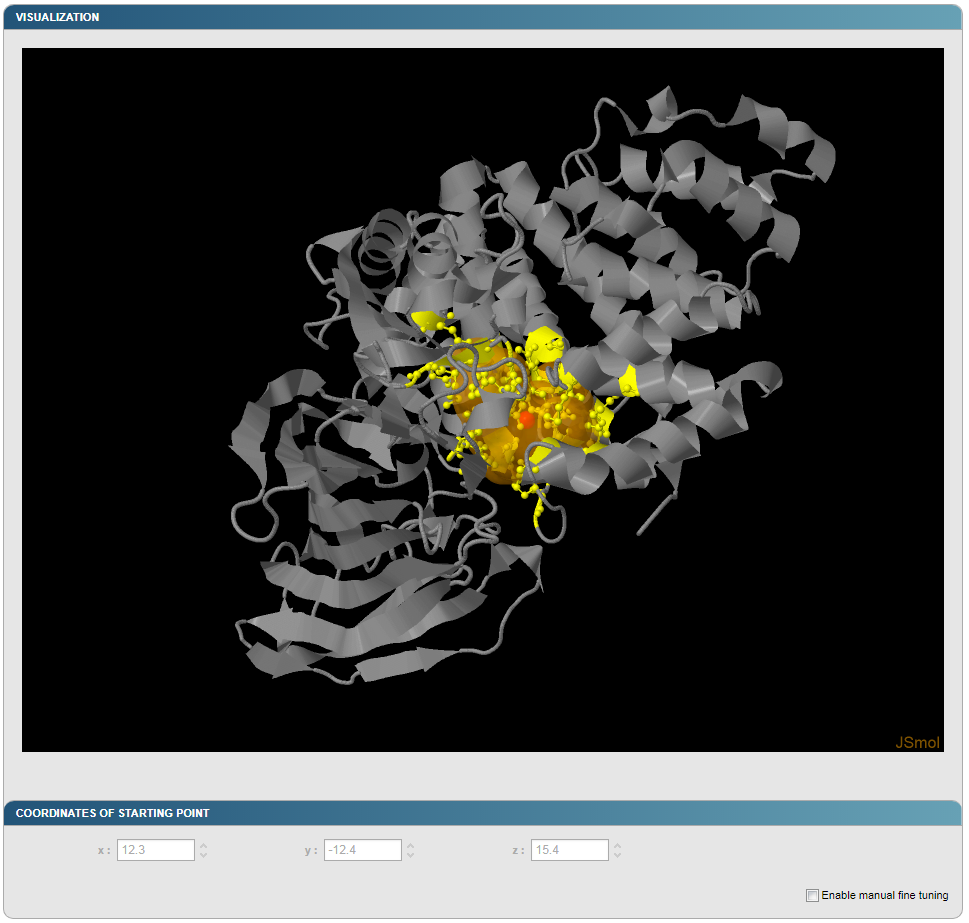


**Figure 4.** The visualization of the selected pocket.

### Setting up the Caver parameters

On “Caver parameters” page the user will determine how the tunnels are calculated (Figure 5). You can select non-canonical amino acid residues to be included on the tunnel calculation. In this tutorial, there is Zn^2+^ ion localized at the site where the natural ligand binds, and therefore the user should select this atom to be included in the tunnel calculation. There is also the inhibitor that the protein was crystalized with, but since it will be in one of the tunnels the user should leave the 1V6 unchecked, as well as the other options, since they are not important for our tunnels. There is the possibility of including a name for the job and an email address to receive notification once the jobs are ready.

The Caver settings in the next form are all changeable, but the standard settings are used in this case tutorial. You will find more information about the individual settings by clicking the “?” icon next to them. On the top right corner of the “Caver settings” window the user can find the Reset values button. The default settings have been determined through extensive testing and they should provide users with reliable data in most cases. If you changed some values, click on reset defaults and then, click on “Submit job”.


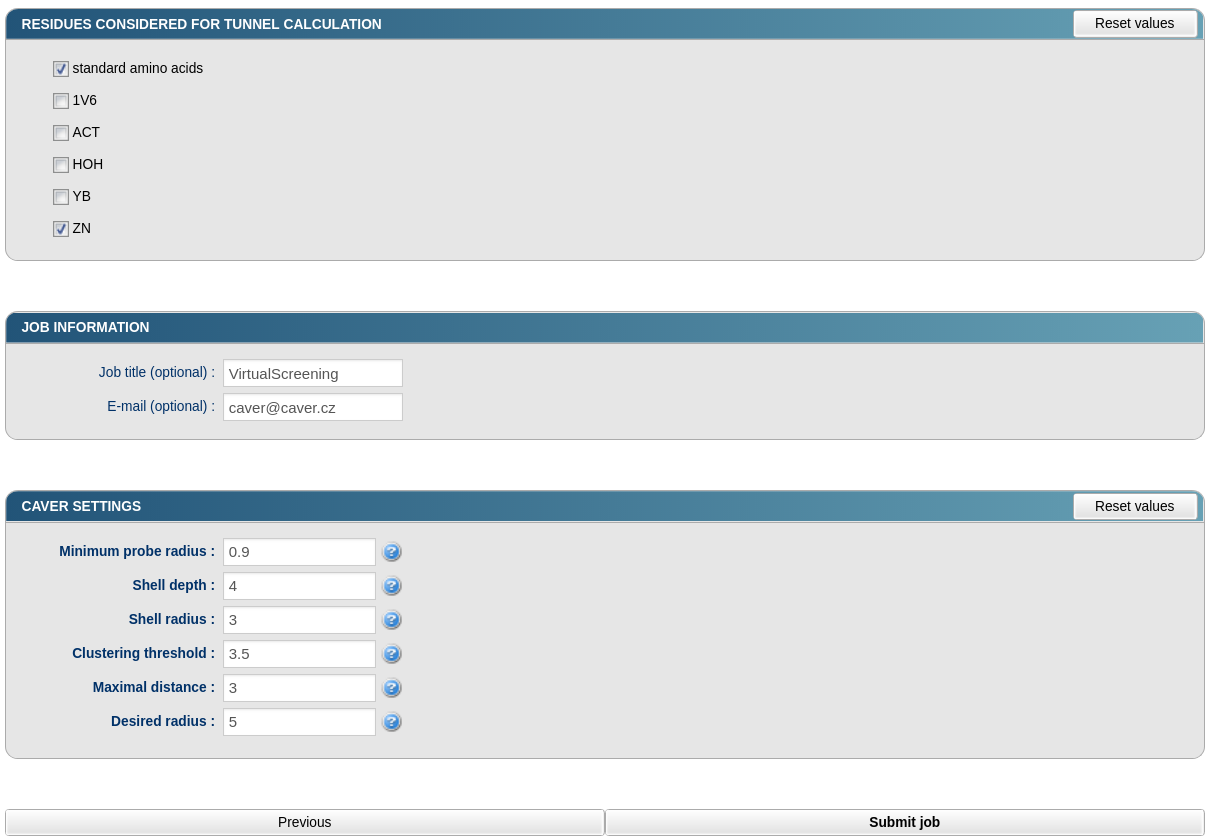


**Figure 5.** Settings for the tunnel calculation parameters.

### Analyzing the tunnels

On the following page, the user has an overview of the tunnel statistics and a window to visualize the found tunnels. If no tunnels are found, the user should go back to the previous page, set a smaller probe size and recalculate the tunnels.

The tunnels info panel shows the general statistics of the found tunnels, such as the bottleneck radius and the length of the tunnel (Figure 6). The small icons at the end of each tunnel let you take a look at the tunnel details, zoom in on the tunnel in the visualization and to see the tunnel profile (the radius vs length graph). From the panel on the right of the page, you can download the visualization of the tunnels as a PyMOL session, all the results as a zip file as well as the configuration and log files.

The visualization settings allow different visualization styles for the protein, the tunnels and the pocket. You can visualize the tunnel as spheres or tracing its center line, and the protein can be presented as cartoon, sticks, wireframe, balls and sticks, trace and backbone. You can also visualize the pocket with the starting point for the tunnel calculation. It is possible to export images directly from the Caver Web interface, just click ono the “Save image” button and you can download the visualized image as a PNG file.

**
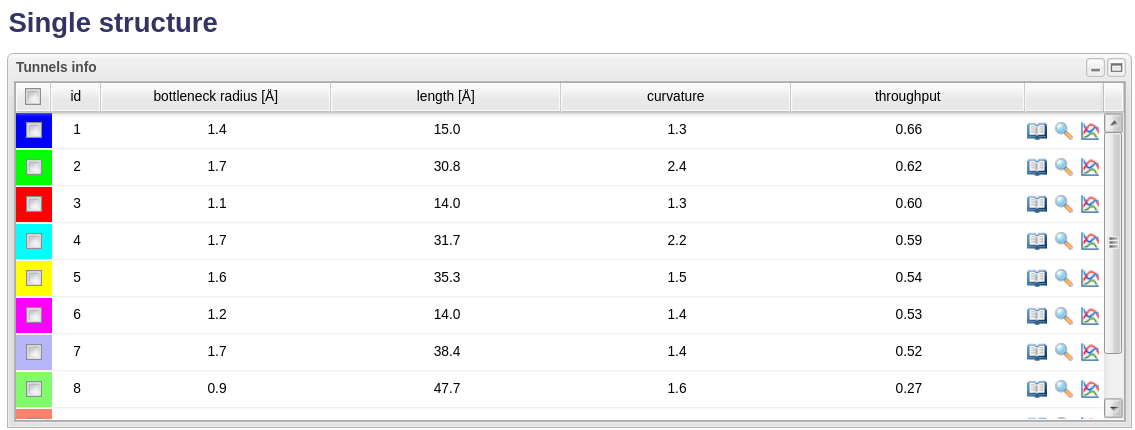
**

**Figure 6.** Window with tunnels info for all the tunnels.

More information on the tunnels may be obtained by pressing the “booklet” icon on the right side of the tunnel. A window will pop up (Figure 7a) with four different options: Overview, Bottlenecks, Centerline and Residue & atoms. The overview shows details of the tunnel statistics with a visualization of the tunnel profile. In the “Bottleneck” tab, you will see the tightest point of the tunnel with the surrounding residues and details of it. The “Centerline and Residues & Atoms” tabs will show you the coordinates for the tunnel centerline and a detailed list of the Residues & Atoms that surround the tunnel, respectively.

The user can also zoom the view of a specific tunnel when pressing the magnifier icon, on the right side of the booklet icon. And the third icon on the Tunnels info window will allow the user to view the Tunnel profiles (Figure 7b). This visualization maybe tunnel by tunnel or as many tunnels together as the user wants. This window may be exported as PNG and CSV so the user can use the figure directly or the values to build their own tunnel profile visualization.

**
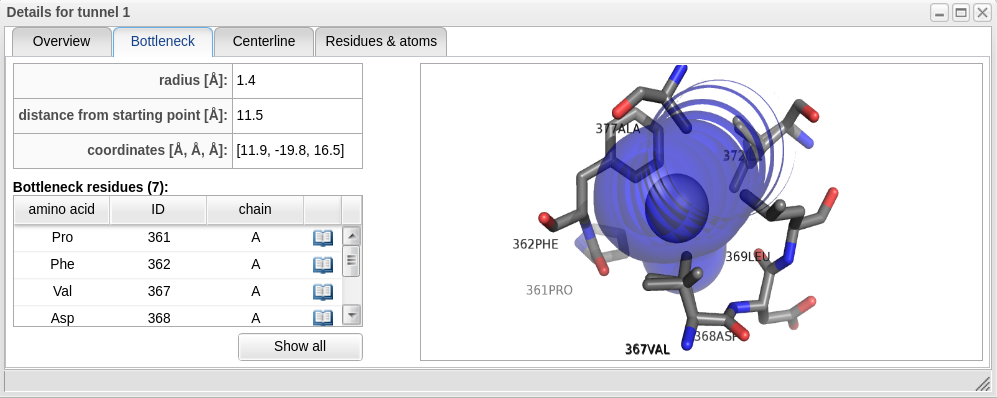
**

**
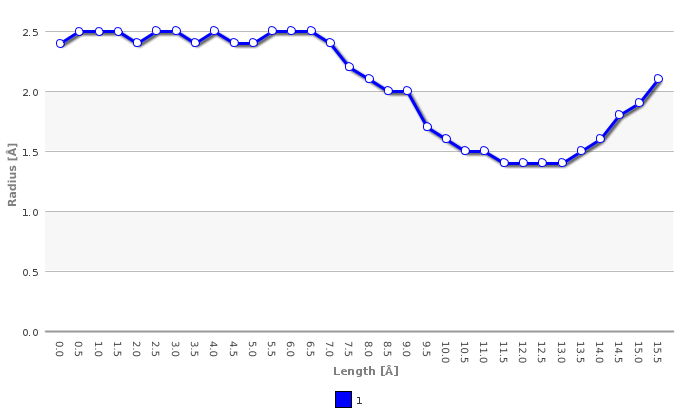
**

**Figure 7.** a) Details of the tunnel used in this case study. Bottleneck shown with the residues creating that bottleneck identified with three letter code and numbering. b) Tunnel profile shown. X-axis shows the length of the tunnel from starting on zero (starting point) and finishing on the bulk solvent.

### 5. Selecting ligands

We are screening for anti-inflammatory drugs to inhibit the inflammatory response promoted by leukotriene A4 hydrolase/aminopeptidase. The tutorial molecules were downloaded from drugbank.ca. You can find them by searching for anti-inflammatory in drugs and filtering only EU approved molecules. We downloaded 21 drugs avoiding antibodies and peptidic structures and uploaded them into the Caver Web (Figure 8). The set of ligands can be downloaded from the following location <https://loschmidt.chemi.muni.cz/caverweb/data/case3_ligands.zip>.

After uploading the ligand structures, the user should press “Process” button. It may take a few minutes before starting the CaverDock calculation. The processing time naturally depends on the number of uploaded ligands.


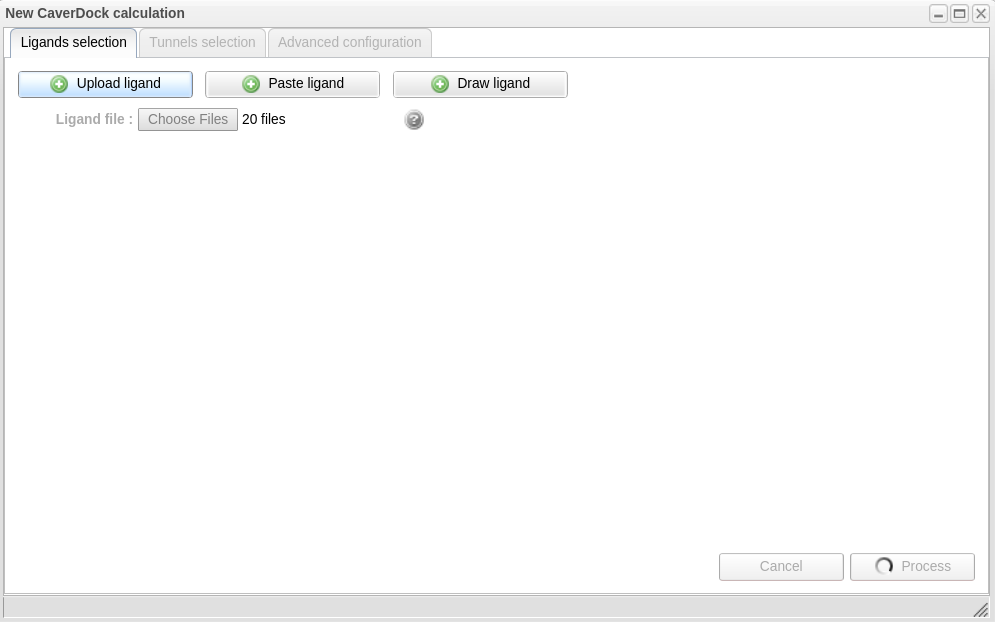


**Figure 8.** Window for starting a new CaverDock calculation. Starting with the Ligands selection the ligands are uploaded. Parameters in the Tunnel selection and the Advance configurations tabs will not be changed in this case study. Default settings are used so tunnel #1 is already chosen.

Once the processing of the ligands is finished, the user chooses the direction in which the calculation should be made, either IN or OUT. The direction chosen in “IN”, which is also the default, since inhibitors are being tested in this tutorial. Do **NOT** press “Submit” yet.

The tunnel with the highest throughput is selected for the CaverDock calculation by default. You can change the tunnel for calculation or add more tunnels on the “Tunnel selection” tab. Remember though, that you can only run 20 jobs simultaneously and each tunnel will be studied with each ligand. There is also a tab for “Advanced configuration” where different parameters for the CaverDock calculation can be changed. We will use the default parameters in this tutorial.

Once you have finished setting up the ligands, tunnels and parameters, you can press “Submit” to start the CaverDock calculations. The CaverDock calculations take approximately 10-60 min depending on the size of the ligand, the size and shape of the tunnel. You can also inspect the pre-calculated results using the ‘pre-calculated results’ link in the Example section on the web.

### Analyzing the data

The ZZZ symbol turns into a green check symbol when the calculations are over. The report from the calculation can be generated. Notice that one of the calculations has not be completed: auranofin. There are two common reasons for non-finishing calculations: (i) the atom types in the ligand are not supported by the AutoDock Vina (e.g., Au in auranofin) or (ii) the ligand could not pass through the specific tunnel due to its size or geometry. We cannot estimate the binding of the ligands containing the atoms, which are not supported by the AutoDock Vina. However, we can estimate that the ligands failing to pass through the tunnels either need a conformational changes of the protein for their binding or they cannot bind to the target site.

You can click on the “book” icon on the Job information panel for the successfully completed calculations (Figure 9a). This opens a “CaverDock results” window which shows the energetic profile of the ligand binding (Figure 9b). Here, you can select the points on the graph corresponding to the bound state (E_bound_), maximum energy barrier (E_max_) and unbound state (E_surface_). Click on the “pencil” icon next to the corresponding fields and click on the corresponding spot on the binding trajectory graph. In this tutorial, the binding site will be on the far right, and the surface on the far left. Caver Web will automatically calculate an estimate of the binding energy barrier (E_A_) and the difference in energy of the bound and unbound states (E_BS_). Once you have saved the values, you can close the window. Repeat this procedure for all the successful calculations to generate the report with the final results.

To generate the final report, the user should press the “Generate report” button on the bottom right side of the “Job information” panel (Figure 9a). A window will pop up with two tabs: “Job selection” and “Energy profile”. For this case we will choose every job and will keep the default options on the Energy profile (Figure 10). A normalization of the plots will help to compare individual inhibitors.


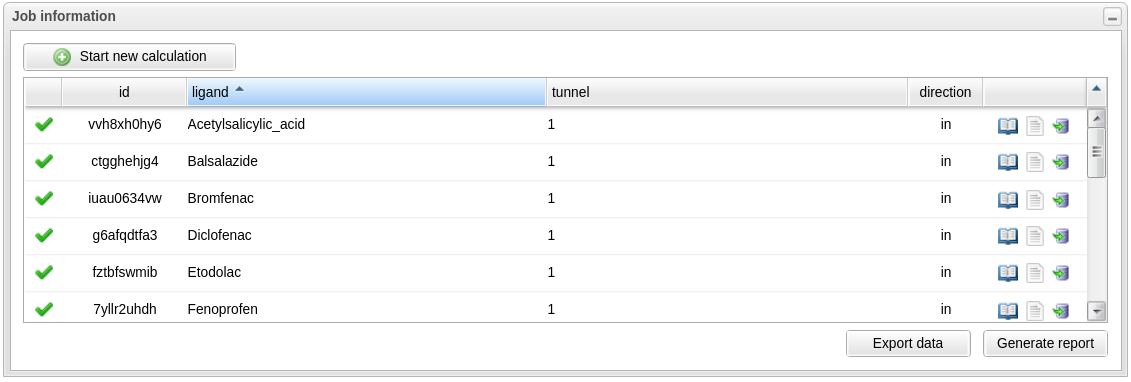


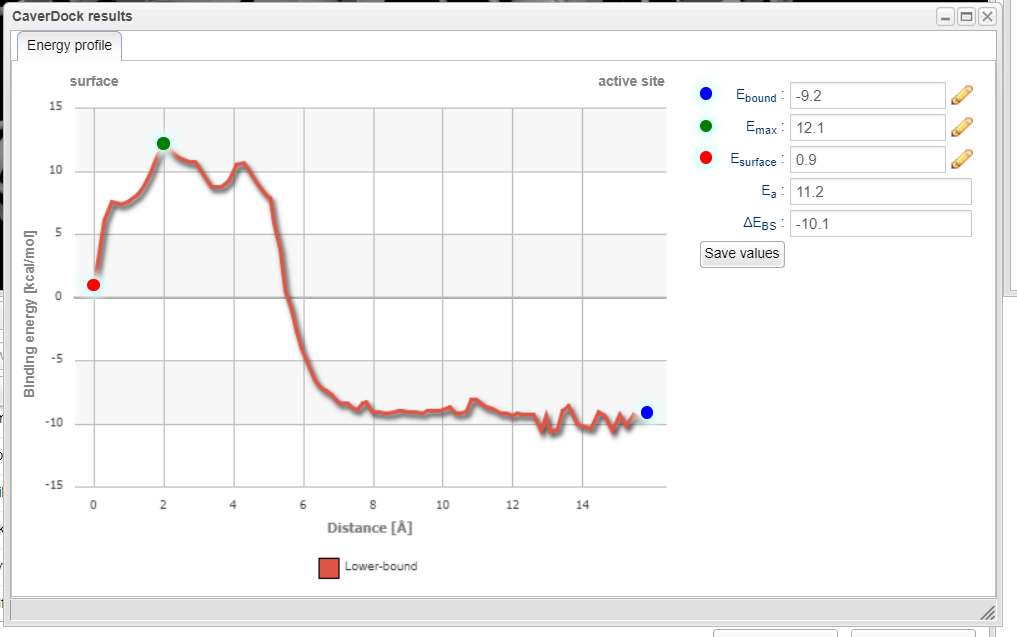


**Figure 9.** a) The Job information panel displaying the state and results of the CaverDock jobs. b) CaverDock results window displaying the energetic profile of the binding.


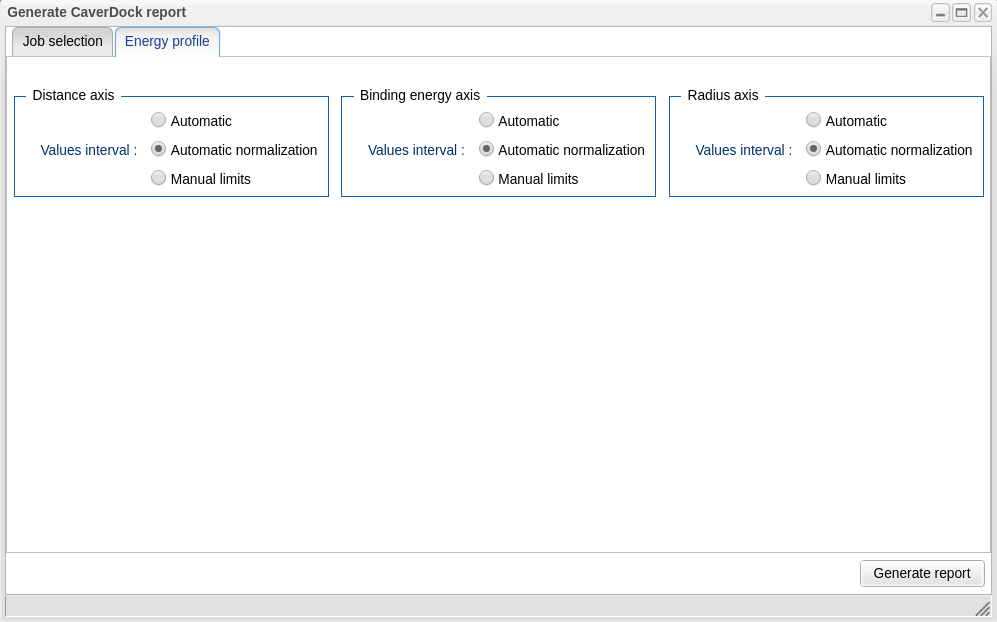


**Figure 10.** Generate report window with selected automatic normalization for comparative analysis.

The generated report is available <https://loschmidt.chemi.muni.cz/caverweb/data/case3_report.pdf>. It starts with basic information about the job, including the PDB id and the generation time of the report. Then the tunnels used in calculations are shown in a table with their characteristics. The second table displays the CaverDock results, including the user defined energetics. On the following pages, the individual energy profiles of the jobs can be inspected.

In this virtual screening, we are interested in the easiness of the access to the druggable site. This property of the ligands can be seen in the energy values E_Max_ (the highest binding energy in the trajectory) and E_a_ (activation energy of association: E_Max_ - E_Surface_ for reactants). To select well binding ligands, we should aim for the lowest values in both of these energy values.

The five lowest E_Max_ values are with the ligands nabumetone, fenoprofen, rabeprazole, balsalazide and ketoprofen. The five lowest E_a_ values are with the ligands flurbiprofen, ibuprofen, ketoprofen, ketorolac and nabumetone. These lists overlap, and by selecting the overlapping ligands we have found the most easily bound ligands which would be ibuprofen and flurbiprofen.

The energy profiles illustrate that most of the ligands have the highest energy barriers at the smallest tunnel radius point around 3Å. It is possible that this part of the enzyme shows dynamic movements during the ligand binding. The analysis enabling the side-chains’ flexibility can be carried out with the stand-alone version of CaverDock and will be integrated to the web version in the future. The energy profiles of the ligands are not identical suggesting that assessment of the binding based on the tunnel radius only would be difficult. Some ligands display the high binding energies even at the wider parts of the tunnel.

Moreover, some of the inhibitors, such as oxaprozin, have lower binding energies at the tunnel instead of the catalytic site. It is possible that this inhibitor preferably binds to the tunnel instead of the active site, thus blocking the access of substrates going in or products going out.

### Conclusions

Analyzing the data obtained from this virtual screening using the Caver Web reveals that the inhibitors ibuprofen and flurbiprofen have the easiest passage through the main tunnel. The data also reveals that oxaprozin binds better inside the access tunnel than in the active site, indicating its possible inhibition mechanism by the tunnel blockage. The structure of this complex and the mechanistic information would not be available from a traditional virtual screening targeting the active site.

### References

1. Banegas-Luna,A.-J., Cerón-Carrasco,J.P. and Pérez-Sánchez,H. (2018) A review of ligand-based virtual screening web tools and screening algorithms in large molecular databases in the age of big data. *Future Med. Chem.*, **10**, 2641–2658.

2. Funk,C.D. (2001) Prostaglandins and leukotrienes: Advances in eicosanoid biology. *Science*, **294**, 1871–1875.

3. Samuelsson,B. (1983) Leukotrienes: mediators of immediate hypersensitivity reactions and inflammation. *Science*, **220**, 568–575.
